# Comparative Proteomic Analysis Reveals Elevated Capacity for Photosynthesis in Polyphenol Oxidase Expression-Silenced *Clematis terniflora* DC. Leaves

**DOI:** 10.3390/ijms19123897

**Published:** 2018-12-05

**Authors:** Xi Chen, Bingxian Yang, Wei Huang, Tantan Wang, Yaohan Li, Zhuoheng Zhong, Lin Yang, Shouxin Li, Jingkui Tian

**Affiliations:** 1 College of Biomedical Engineering & Instrument Science, Zhejiang University, Zheda Road 38, Hangzhou 310027, China; 21615052@zju.edu.cn (X.C.); xianyb@zju.edu.cn (B.Y.); 21615051@zju.edu.cn (W.H.); 21615085@zju.edu.cn (T.W.); 21815031@zju.edu.cn (Y.L.); zhongzhh@zju.edu.cn (Z.Z.); 2 Zhuhai Weilan Pharmaceutical Co., Ltd., Zhuhai 519030, China; LinYangzhuhai@163.com; 3 Changshu Qiushi Technology Co., Ltd., Suzhou 215500, China; 0016837@zju.edu.cn; 4 Zhejiang-Malaysia Joint Research Center for Traditional Medicine, Zhejiang University, Hangzhou 310027, China

**Keywords:** *Clematis terniflora* DC., polyphenol oxidase, virus induced gene silencing, photosynthesis, glycolysis

## Abstract

Polyphenol oxidase (PPO) catalyzes the o-hydroxylation of monophenols and oxidation of o-diphenols to quinones. Although the effects of PPO on plant physiology were recently proposed, little has been done to explore the inherent molecular mechanisms. To explore the in vivo physiological functions of PPO, a model with decreased PPO expression and enzymatic activity was constructed on *Clematis terniflora* DC. using virus-induced gene silencing (VIGS) technology. Proteomics was performed to identify the differentially expressed proteins (DEPs) in the model (VC) and empty vector-carrying plants (VV) untreated or exposed to high levels of UV-B and dark (HUV-B+D). Following integration, it was concluded that the DEPs mainly functioned in photosynthesis, glycolysis, and redox in the PPO silence plants. Mapman analysis showed that the DEPs were mainly involved in light reaction and Calvin cycle in photosynthesis. Further analysis illustrated that the expression level of adenosine triphosphate (ATP) synthase, the content of chlorophyll, and the photosynthesis rate were increased in VC plants compared to VV plants pre- and post HUV-B+D. These results indicate that the silence of PPO elevated the plant photosynthesis by activating the glycolysis process, regulating Calvin cycle and providing ATP for energy metabolism. This study provides a prospective approach for increasing crop yield in agricultural production.

## 1. Introduction

Polyphenol oxidase (PPO) is an oxidoreductase that catalyzes the oxidation of monophenols and/or o-diphenols to o-quinones, which form brown melanin pigments in fruits and vegetables by covalently modifying and cross-linking proteins [1,2]. However, PPO is not only involved in the formation of pigments, but also plays a crucial role in the biosynthesis of secondary metabolites such as aurones [3] and betalins [4]. PPO solitarily catalyzes the hydroxylation and oxidative cyclization of chalcones, leading to the formation of aurone [3]. In addition, PPO, a tyrosinase can also hydroxylate tyramine into dopamine, which in the presence of betalamic acid yields dopamine-betaxanthin that can be further oxidized to yield 2-des-carboxy-betanidin [4]. Furthermore, PPO also has roles in plant defense against insects and pathogens. In transgenic tomato plants, PPO overexpression greatly increases resistance to *Pseudomonas syringae* [5], and the manipulation of PPO activity provides simultaneous resistance to both disease and insect pests [6]. A recent study on strawberries illustrated that PPO overexpression delays the fungal infection [7]. PPOs may perform different functions in diverse plant species and possibly have multiple roles in plants for the huge PPO gene families.

Potential roles for PPO in plants have also been suggested for adaption to abiotic stresses. A study in olive trees showed oxidation of phenolic is inhibited by decreased PPO, suggesting there is an association between decreased PPO activity and improved antioxidant capacity [8]. The role of PPO in regulation of cell death in walnuts was explored, where silencing of PPO resulted in increased tyramine, which elicited cell death in walnuts [9]. Furthermore, a recent study also demonstrated the potential of PPO in bioremediation and food/drug industries as it significantly reduces the phenol content in an artificial solution [10]. As well as evidence supporting that PPOs play a role in plant defense against biotic stressors, several independent lines of evidence identified PPO with a chloroplastic location, linking PPO with photosynthesis. However, although an interaction between photosynthesis and PPO activity has been presented more than once [11,12,13], evidence either for or against direct involvement has been ambiguous, and the absence of chloroplastic substrates that were identified remains an issue.

Virus-induced gene silencing (VIGS) takes advantage of plant RNAi-mediated antiviral defense and has been used widely in plants to analyze gene function [14]. Lee et al. [15] used VIGS to analyze the susceptibility and resistance functional wheat genes involved in *Zymoseptoria tritici*. Using the barley stripe mosaic virus—VIGS technique, Zhao et al. [16] indicated that the TNBL1 gene is an important gene positively involved in wheat defense response to barley yellow dwarf virus infection. Groszyk et al. [17] analyzed the Bx1 gene ortholog in rye using VIGS and found it to be functionally involved in benzoxazinoid biosynthesis. Recently, VIGS has been a powerful alternative technology for determining the unknown functions of genes and a combination of VIGS and omics is evolving as a competitive strategy for gene function analysis. VIGS and proteomic analysis of the resistance of cotton to *Veticillium dahia* revealed that gossypol, brassinosteroids, and jasmonic acid contribute to this process [18]. The role of the tomato TAGL1 gene in regulating the accumulation of fruit metabolites was investigated using VIGS and metabolomics analyses [19]. An improved virus-induced gene silencing approach was used to elucidate the role of highly homologous *Nicotiana benthamiana* ubiquitin enzymes gene family members in plant immunity [20]. In addition, VIGS technology as a powerful investigation tool is still on the way with its application potentials remaining to be fully developed.

*Clematis terniflora* DC. is a Chinese folk medicinal resource with important pharmaceutical value in the treatment of inflammatory symptoms in the respiratory and urinary systems [21]. Studies have shown that the extracts from the leaves and stems of *C. terniflora* have anti-inflammatory, anti-tumor, and anti-nociceptive effects [22,23,24,25]. Furthermore, omics technologies were used on *C. terniflora* to prospectively understand the inherent mechanism underlying its medicinal quality [24,26]. Considering the role PPO played in stress resistance of plant and biosynthesis of secondary metabolites, regulating PPO activity or gene expression can be beneficial to the yields and quality of medicinal plants. Therefore, the potential relationships between photosynthesis and PPO activity are highly relevant to the improvement of yields and quality [27]. To have insight into this relationship, a comparative proteomic analysis was performed on the leaves of *C. terniflora* DC. with down-regulated PPO activity by VIGS in this study. High-intensity UV-B and dark incubation (HUV-B+D), which are helpful stressors for medicinal plants [28], were used for an integration study.

## 2. Results

### 2.1. Cloning, Sequence Analysis, and Phylogenetic Tree Analysis of CtPPO

Following the identification of CtPPO transcript in *C. terniflora* transcriptome data, 1681 bp of PPO cDNA was cloned from *C. terniflora* leaves using RT-PCR and 5′ RACE with the complementary application of genome-walking technologies (Figure S2a). Sequence analysis of CtPPO cDNA revealed a 166-bp 5′-untranslated region (UTR) located upstream of a start codon and an open reading frame (ORF) of 1681 bp encoding 586 amino acids with a calculated molecular mass of 66.11 kDa and an isoelectric point of 7.18.

Blast analysis of the predicted CtPPO amino acid sequence revealed 62% similarity with *Nelumbo nucifera*. To further understand the evolutionary relationships between CtPPO and PPO in other plants, a phylogenetic tree was constructed based on amino acid sequence. Phylogenetic analysis showed CtPPO has a close evolutionary relationship with the PPO gene in *N. nucifera*, but not other plant species (Figure S2b).

### 2.2. Virus-Induced Gene Silencing of CtPPO in C. terniflora DC

**Figure 1:**
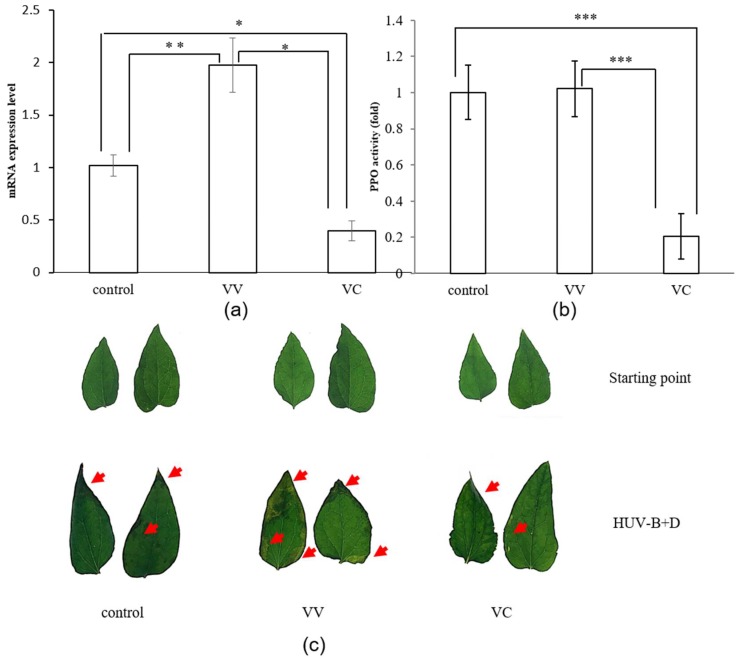
(**a**) Expression levels of CtPPO gene mRNA in leaves of *C. terniflora* in control, VIGS-vector, and VIGS-CtPPO plants. Data are shown as mean ± SD of the independent biological replicates. Asterisks indicate significant changes as measured by Student’s *t*-test (* *p* < 0.05, **p < 0.01, and ***p < 0.001); (**b**) PPO activity was assayed in *C. terniflora* leaves of control, VIGS- vector and VIGS-CtPPO plants. Data are shown as mean ± SD of independent biological replicates. Asterisks indicate significant changes as measured by Student’s *t*-test (* *p* < 0.05, ** *p* < 0.01, and *** *p* < 0.001); (**c**) Morphology of control, VIGS-vector, and VIGS-CtPPO *C. terniflora* plants before and after HUV-B+D treatment. Photograph showing *C. terniflora* growth under different treatments. The damaged areas were marked with red arrows.

VIGS provides an alternative approach for functional analysis of genes. Here, to determine the role of CtPPO, we silenced CtPPO using tobacco rattle virus (TRV)-mediated gene silencing. We constructed two modified TRV vectors: TRV-CtPPO and TRV-PDS. To determine the extent of silencing, we performed qRT-PCR to analysis the transcript levels of the CtPPO gene in inoculated plants. RNA extracted from *C. terniflora* agro-infiltrated with empty vector TRV or TRV-CtPPO or control leaves 20 days post-infection were used for qRT-PCR. As shown in Figure 1a, CtPPO expression levels were reduced in plants infiltrated with TRV-CtPPO compared to the control plants (VIGS-vector and control). To further confirm CtPPO expression, we measured CtPPO activity in control, TRV-vector and TRV-CtPPO plants. In VIGS-CtPPO (VC), PPO activity was significantly lower than in VIGS-vector (VV) and control, illustrating that a successful VC plant model was constructed (Figure 1b). Despite these obvious changes in CtPPO in plants, however, there were no macroscopic phenotypic differences in the VC, VV, and control leaves at the starting point (Figure 1c).

### 2.3. Effects of VIGS-CtPPO and VIGS-Vector on Leaf Proteins in C. terniflora DC.

**Figure 2:**
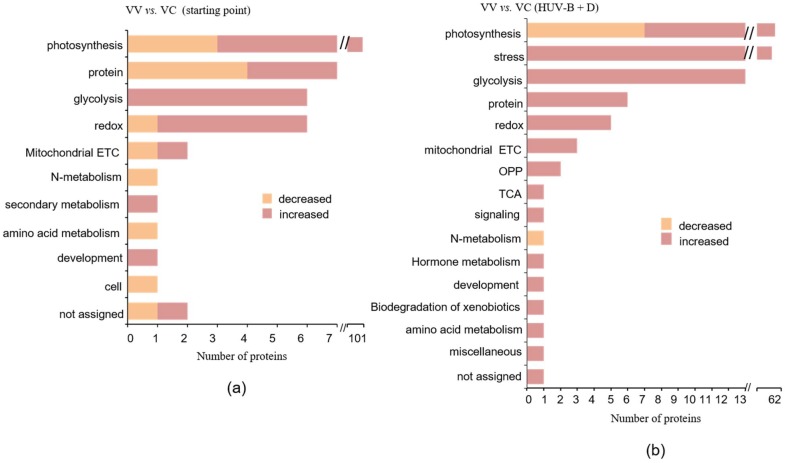
(**a**,**b**) Functional distribution of proteins identified in VIGS-vector and VIGS-CtPPO *C. terniflora* DC. leaves. The leaves were collected at the (**a**) starting point and (**b**) after HUV-B treatment. Proteins were extracted, reduced, alkylated, digested, and analyzed by nanoLC-MS/MS. Protein content is reported as mol %. Protein functions were predicted and categorized using MapMan bin codes. Abbreviations: cell, cell division/organization/cycle; TCA, tricarboxylic acid; ETC, electron transport chains; OPP, oxidative pentose phosphate.

To investigate the effects of CtPPO on *C. terniflora* leaf proteins, a gel-free/label-free proteomic technique was used. Proteins extracted from VC- and VV-infected *C. terniflora* leaves were reduced, alkylated, digested, and analyzed by nano-LC-MS/MS. The obtained proteomic data were analyzed using the UniProtKB/Swiss-Prot database and protein content was estimated by mol%. The functions of identified proteins were predicted based on comparisons with functional annotations of the *Arabidopsis* genome and classified using MapMan bin codes. The results of the functional analyses demonstrated that the proteins with increased expression were mainly enriched in photosynthesis, glycolysis, redox, protein metabolism, and secondary metabolism, while the proteins with decreased expression were mainly enriched in protein metabolism and photosynthesis. Notably, expression of proteins related to secondary metabolism, development, and glycolysis were increased in the VC plants, whereas the expression of proteins related to N-metabolism, amino acid metabolism, and cell were decreased (Figure 2a; Tables S8 and S9).

### 2.4. Effects of High-Level UV-B and Dark Treatment on Proteins in VIGS-CtPPO and VIGS-Vector C. terniflora DC. Leaves

HUV-B+D dramatically impacts plant physiology [24]. To further investigate the effects of CtPPO on *C. terniflora*, we exposed VC, VV, and control plants to HUV-B+D. Subsequent phenotypic analysis revealed the leaves to be wilted with burned patches and a high degree of crispation in control plants. The leaves of VV plants were similar to the control plants, while the leaves from the VC plants appeared significantly less damaged than in the control and VV plants (Figure 1c). To determine the expression profiles of leaf proteins in VC and VV after HUV-B+D, a gel-free/label-free proteomic approach was used. Proteins extracted from VC and VV plant leaves after HUV-B+D were reduced, alkylated, digested, and analyzed by nano-LC-MS/MS. Obtained proteomic data were analyzed using the UniProtKB/Swiss-Prot database and the protein content was estimated by mol%. The functions of identified proteins were predicted based on comparisons with functional annotations of the *Arabidopsis* genome and classified using MapMan bin codes. The results of the functional analyses demonstrated that HUV-B+D increased expression of proteins involved in most of the functional classes, including photosynthesis, stress, glycolysis, protein metabolism, and redox, in VC plants compared to VV plants. However, expression of proteins involved in N-metabolism was decreased in VC plants compared to VV plants (Figure 2b).

### 2.5. Integrated Analysis of Proteins in VIGS-CtPPO and VIGS-Vector C. terniflora Differentially Expressed after HUV-B+D

The proteins identified in leaves collected from VC and VV plants at the starting point were 676 and 662, respectively. Among the identified proteins, 480 and 467 proteins were commonly expressed in VV and VC plants, respectively, at the starting point and after HUV-B+D. Alternatively, there were 443 and 455 proteins commonly expressed at the starting point and HUV-B+D in VV and VC plants, respectively (Figure S3).

**Figure 3:**
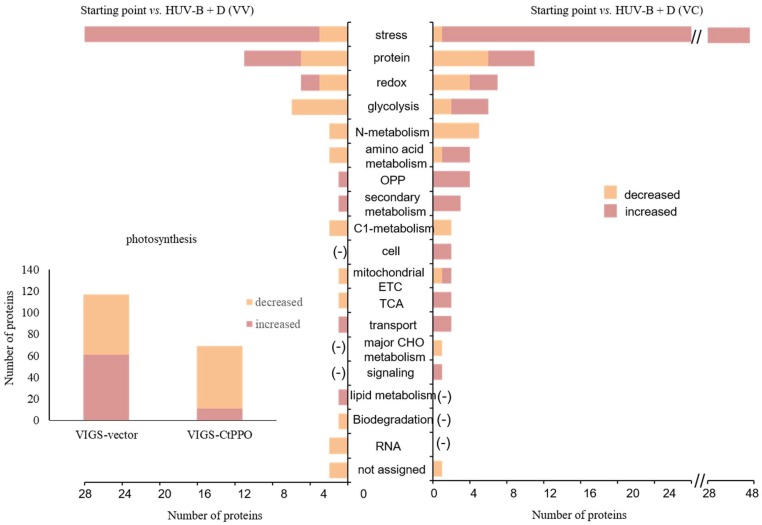
Functional distribution of proteins identified in VIGS-vector and VIGS-CtPPO *C. terniflora* DC. leaves. Leaves were collected at the starting point and after HUV-B+D treatment. Proteins were extracted, reduced, alkylated, digested, and analyzed by nanoLC-MS/MS. Protein content is reported as mol %. Protein functions were predicted and categorized using MapMan bin codes. Abbreviations: cell, cell division/organization/cycle; TCA, tricarboxylic acid; ETC, electron transport chains; CHO, carbohydrate; OPP, oxidative pentose phosphate; (-), not determined.

For proteins differentially expressed in response to HUV-B+D in VV and VC plants, further integration analysis was performed. Figure 3 shows that the number of proteins involved in photosynthesis displaying increased expression was decreased in VC plants compared with that in VV plants. The number of increased proteins related to stress, OPP, secondary metabolism, and transport was increased in VC plants compared to VV plants. The number of decreased proteins associated with stress, glycolysis, amino acid metabolism, and carbon-one(C1)-metabolism was decreased in VC plants compared to VV plants. Furthermore, proteins involved in glycolysis, amino acid metabolism, cell, mitochondrial ETC, TCA, and signaling displaying increased expression were only detected in VC plants (Figure 3, Tables S6 and S7).

### 2.6. MapMan Analysis of the VIGS-CtPPO and VIGS-Vector C. terniflora Leaf Proteomic Data at the Starting Point and after HUV-B+D

**Figure 4:**
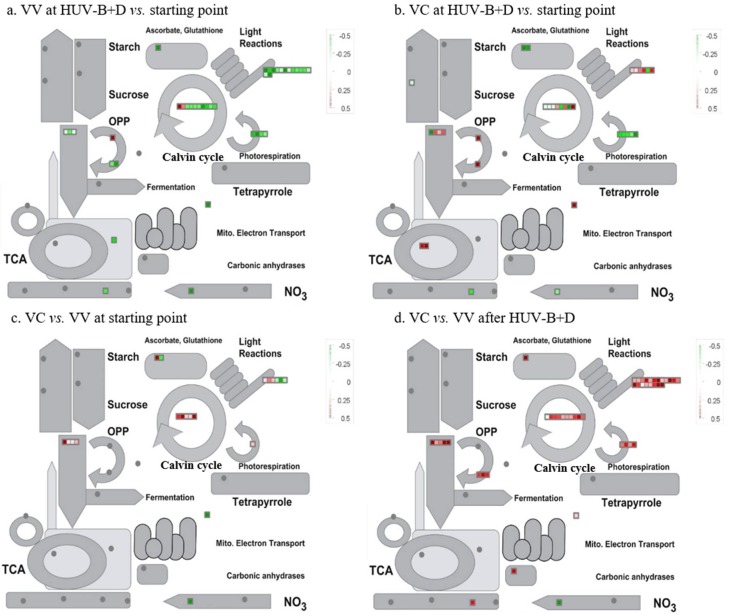
Metabolic pathways of proteins identified in VIGS-vector and VIGS-CtPPO *C. terniflora* DC. Leaves were collected at the starting point and after HUV-B+D treatment. The proteins were grouped into functional categories related to primary metabolism and changes in abundance were visualized using MapMan software. Each square and color indicates the fold-change of a differentially expressed protein. Green and red indicate a decrease and increase, respectively, in fold change compared with the corresponding group: (**a**) comparison of proteins in VIGS-vector leaves after HUV-B+D treatment at starting point, (**b**) comparison of proteins in VIGS-CtPPO leaves after HUV-B+D treatment at starting point, (**c**) comparison of proteins at starting point in VIGS-CtPPO and VIGS-vector plants, and (**d**) comparison of proteins after HUV-B+D treatment in VIGS-CtPPO and VIGS-vector plants. Abbreviations: OPP, oxidative pentose phosphate; CHO, carbohydrate; TCA, tricarboxylic acid cycle.

To examine changes in the levels of the identified proteins in-depth in VV and VC plants, the DEPs were analyzed using MapMan software. The analysis identified the main functional categories of the proteins displaying significant changes in expression as involved in the light reaction, photorespiration, and Calvin cycle. HUV-B+D conditions induced dramatic decrease in Calvin cycle and light reaction related proteins in VV, however, very interestingly, it was significantly rescued by the silencing of CtPPO in VC. Further analysis showed that the silencing of CtPPO led to the increase of Calvin cycle related proteins in VC than VV at starting point (Figure 4a,b). After exploring HUV-B+D, the silencing of CtPPO gave a further increase of Calvin cycle related proteins. Additionally, it also resulted in the synchronously increase on light reaction and photorespiration related proteins in VC than VV at HUV-B+D (Figure 4c,d).

Following this preliminary analysis, we further studied the differences in enzymes in the light reaction and Calvin cycle. The proteins involved in the light reaction and Calvin cycle was mostly decreased in VV but there was no obvious change in VC after HUV-B+D treatment. At starting point, 1 and 3 proteins in photosystem I and II of light reaction was increased in VC compared to VV. In Calvin cycle, 3 and 2 proteins involved in carboxylation and reduction was increased in VC compared with VV. However, after HUV-B+D treatment, 19 proteins related to NADP^+^-dependent aldehyde reductase, ATP synthase, and glycerol phosphate dehydrogenase in light reactions was increased and 11 proteins related to the enzymes in reduction and regeneration of Calvin cycle was increased in comparison of VC and VV (Figure S4).

### 2.7. Expression Profile Analysis of Glycolysis-Related Proteins in C. terniflora DC. Leaves

**Figure 5:**
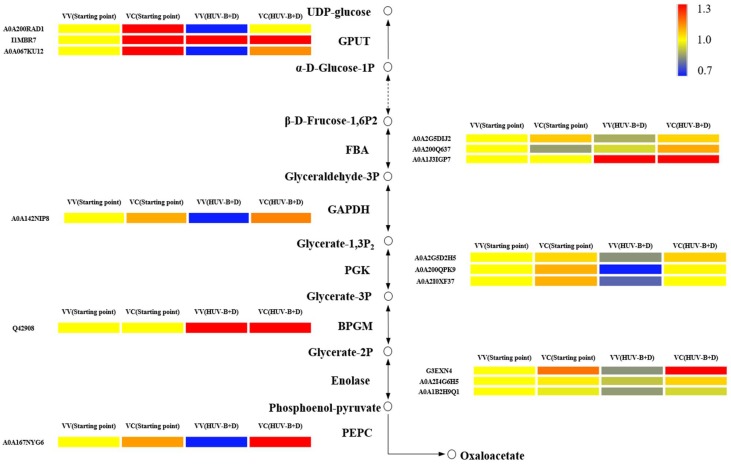
Expression profile analysis of glycolysis-associated proteins. Each square and color indicates the fold-change for a differentially expressed protein. Blue and red indicate a decrease and increase, respectively, in fold-change compared with the corresponding group: VV(SP) proteins in VIGS-vector plants at starting point, VC(SP) proteins in VIGS-CtPPO plants at starting point, VV(HUV-B+D) proteins in VIGS-vector plants after HUV-B+D treatment, and VC(HUV-B+D) proteins in VIGS-CtPPO plants after HUV-B+D treatment. FBA: fructose-1,6-bisphosphate aldolase; GAPDH: glyceraldehyde-3-phosphate dehydrogenase; PEPC: phosphoenolpyruvate carboxylase; circle: metabolite; arrow: direction; solid line: single step; dotted line: multiple step.

Glycolysis related proteins were significantly changed by the silencing of CtPPO. To further characterize the relationship between glycolysis and CtPPO, expression profile analysis of glycolysis-related proteins in *C. terniclora* leaves was conducted. The abundances of glyceraldehyde-3-phosphate dehydrogenase (GAPDH), UTP-glucose-1-phosphate uridylytransferase (GPUT), phosphoglycerate kinase (PGK), phosphoenolpyruvate carboxylase (PEPC), and enolase were increased in VC plants compared to VV plants at the starting point. Moreover, after HUV-B+D, the abundances of GPUT, fructose-1,6-bisphosphate aldolase (FBA), GAPDH, PGK, BPGM, enolase, and PEPC were all increased in VC plants compared to VV plants (Figure 5).

### 2.8. Effects of VIGS-CtPPO on Photosynthesis Characteristics in C. terniflora DC. Leaves

**Figure 6:**
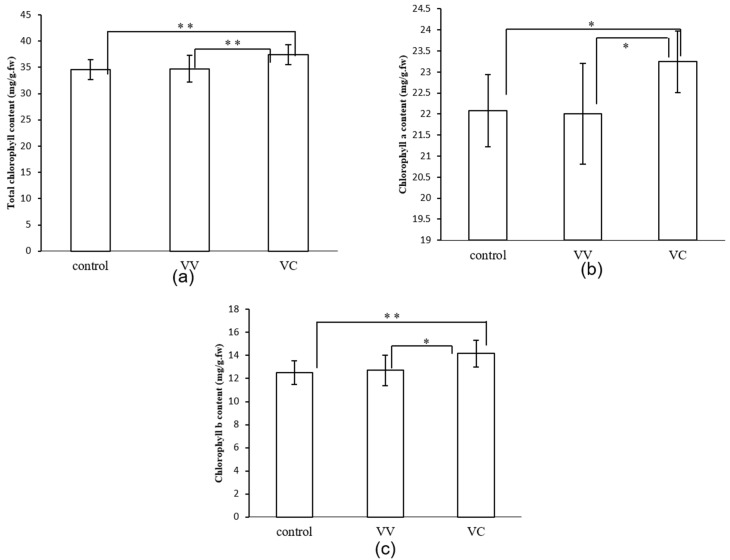
Chlorophyll content in in control, VIGS- vector and VIGS-CtPPO *C. terniflora* leaves. (**a**) Total chlorophyll content, (**b**) chlorophyll a content, and (**c**) chlorophyll b content. Data are shown as mean ± SD for independent biological replicates. Asterisks indicate significant changes as measured by Student’s *t*-test (* *p* < 0.05, ** *p* < 0.01). fw: fresh weight.

To confirm the relationship between CtPPO and photosynthesis, the chlorophyll content was measured first. The results showed that the chlorophyll a, chlorophyll b, and total chlorophyll contents were all higher in VC plants than in VV and control plants (Figure 6). Then the photosynthesis characteristics were measured by using a Li-6400 Portable Photosynthesis System.

**Figure 7:**
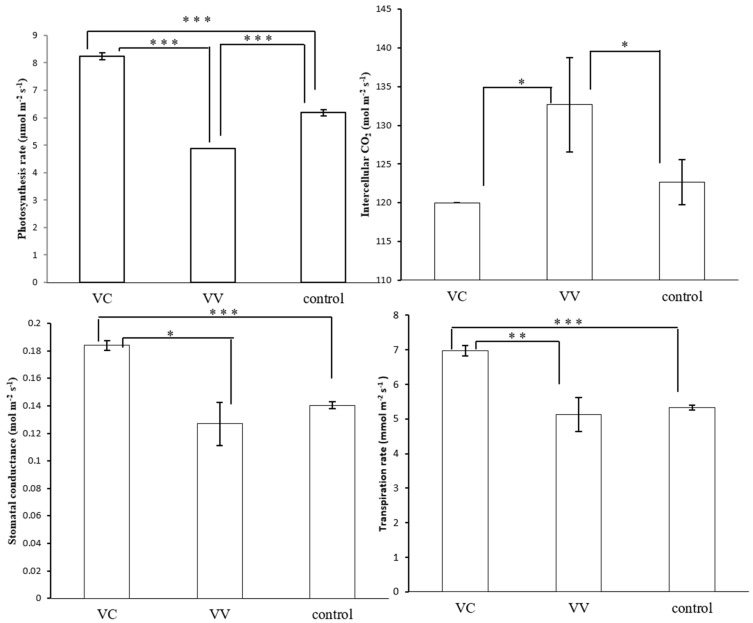
Analysis of photosynthesis characteristics in control, VIGS-vector, and VIGS-CtPPO at starting point. The photosynthesis rate, intercellular CO_2_, stomatal conductance, and transpiration rate were measured using an open gas-exchange system. Data are shown as mean ± SD of independent biological replicates. Asterisks indicate significant changes as measured by Student’s t *t*-test (* *p* < 0.05, ** *p* < 0.01, and *** *p* < 0.001).

The photosynthesis rate, intercellular CO_2_, stomatal conductance, and transpiration rate were further measured in the study. The photosynthesis rate, transpiration rate, and stomatal conductance in VC plants were higher than in VV plants, while the intercellular CO_2_ was lower than that in VV (Figure 7). After HUV-B+D, the damage of photosynthesis rate in VC plants was significantly lower than in VV and control plants, while the stomatal conductance and transpiration rate were low in control, VV, and VC plants. The intercellular CO_2_ was still lower in VC and control plants than in VV plants, but both were higher than that at the starting point (Figure S5). The photosynthesis rate was dramatically decreased in by HUV-B+D in *C. terniflora,* however, it seems that the silencing of CtPPO has a mitigating effect to the damage degree in VC (Figure 7 and Figure S5).

### 2.9. Effects of VIGS-CtPPO on ATP Synthase in C. terniflora DC. Leaves

**Figure 8:**
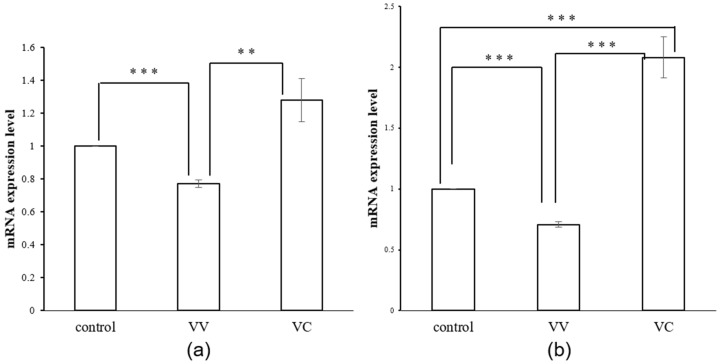
Levels of chloroplast ATP synthase mRNA in control, VIGS-vector, and VIGS-CtPPO *C. terniflora* leaves. (**a**,**b**) *C. terniflora* leaves at the (**a**) starting point and (**b**) after HUV-B+D treatment. Data are shown as mean ± SD of independent biological replicates. Asterisks indicate significant changes as measured by Student’s *t*-test (** *p* < 0.01, and *** *p* < 0.001).

To confirm the relationship between CtPPO and energy metabolism, qRT-PCR-based analysis of ATP synthase was performed. Leaves of VV and VC were collected at starting point and after HUV-B+D. At the starting point, the expression of ATP synthase was increased without statistical significance in VC compared with control and VV (Figure 8). However, after HUV-B+D, ATP synthase was increased by 2-folds in VC plants compared with VV and control plants (Figure 8). These results indicate that the silencing of CtPPO might activate the energy metabolism by up-regulating the ATP synthase in response to HUV-B+D.

## 3. Discussion

### 3.1. Silencing of PPO Promoted the Light Reaction in C. terniflora

The photosynthetic process in plants can operate through linear or cyclic electron flow, which involves three major complexes of the electron transfer chain: photosystem II (PSII), photosystem I (PSI), and cytochrome b6f complex [29,30]. Light reactions are an important step in photosynthesis and can transfer light energy to ATP and NADPH [31]. The initial step in this process is the absorption of light energy by the chlorophyll molecule. Ferredoxins were extensively employed as electron shuttles by anaerobes long before the advent of oxygenic photosynthesis [32], while the ferredoxin-NADP^+^ reductase catalyzes an electron-hydride exchange between reduced ferredoxin and NADP^+^ to yield NADPH [33]. The activation state of chloroplast ATP synthase is regulated by proton-motive forces generated by photosynthetic electron transfer reactions and reduction of disulphides in the γ subunit by TRX [34,35]. A lower ATP content resulting from the loss of ATP synthesis can inhibit the synthesis of ribulose biphosphate and influence the photosynthetic assimilation of CO_2_ [36]. In our study, the amounts of chlorophyll a and chlorophyll b were increased in VC plants compared with the VV and control plants, indicating the silencing of CtPPO could upregulate the PSs system in photosynthesis. In addition, the expression of ATP synthase, and ferredoxin-NADP^+^ reductase were decreased in response to HUV-B+D (Table S6), indicating the inhibition effect of HUV-B+D on *C. terniflora*. However, the expression of ATP synthase (Figure 8) and ferredoxin-NADP^+^ reductase (Table 1) were dramatically increased in VC plants compared with VV plants after HUV-B+D. These results indicate that VIGS of CtPPO had a positive effect on upregulating the light reactions to provide ATP and NADPH for plants under stress.

### 3.2. Silencing of PPO Activated the Calvin Cycle in C. terniflora

The Calvin cycle is an essential carbon fixation process in photosynthesis that converts carbon dioxide to glucose, accompanied by reduction reactions and ribulose 1,5-bisphosphate (RuBP) regeneration [37]. The Calvin cycle is redox-activated process, and the redox homeostasis is mediated by reducing power from photosynthetic electron transport to ferredoxin (Fd) and NADPH, via Fd-thioredoxin (TRX) reductase (FTR) and NADPH-dependent TRX reductase (NTRC) [38]. FTR and multiple TRXs consist the Fd-TRX system, while the NTRC contains a complete TRX system in a single polypeptide. Both systems have an impact on activation of Calvin cycle-related enzymes, including fructose-1,6-bisphosphatase (FBPase), sedoheptulose-1,7-bisphosphatase (SBPase), phosphoribulokinase (PRK), and GAPDH [39,40,41]. In our current study, Calvin cycle-related PGK, GAPDH, PRK, TK, and RuBP were decreased in response to HUV-B+D, indicating HUV-B+D significantly inhibited the Calvin cycle. However, the drop in CtPPO activity led to a dramatic increase in Calvin cycle-related proteins in *C. terniflora* at the starting point and after HUV-B+D. The redox-related proteins were further analyzed and TRX was found to be increased in VC plants compared with VV plants at the starting point (Table 2) and Fd-NADP reductase was increased in VC plants compared to VV plants after HUV-B+D (Table 1). As PPO is located in chloroplast [42,43], these results indicate VIGS of CtPPO can protect the Calvin cycle by activating the FTR and NTRC system.

Chlorophyll biosynthetic enzymes glutamyl-tRNA reductase, Mg-protoporphyrin IX monomethylester cyclase, and plastidic 2-Cys PRXs are direct substrates of NTRC in *Arabidopsis* [44]. As a result, NTRC protects the formation of chlorophyll in *Arabidopsis* [45]. The NTRC, together with 2-Cys PRXs, forms a two component peroxide detoxifying system that acts as a reductant under stress conditions [44]. Overexpression of NTRC raises the CO_2_ fixation rate and lowers non-photochemical quenching by enhancing the activation of TRX-regulated enzymes and chloroplast ATP synthase in the Calvin cycle [46]. In this study, the amounts of total chlorophyll, chlorophyll a, and chlorophyll b were increased in VC plants compared with VV and control plants (Figure 6). The amount of 2-Cys peroxiredoxin was decreased in VV plants in response to HUV-B+D (Table S6), however, it was increased in VC plants compared with VV plants after HUV-B+D (Table 1). Furthermore, chloroplast ATP synthase was increased in VC plants compared with VV and control plants at the starting point and were increased more significantly in VC plants than in VV and control plants following HUV-B+D (Figure 8). The intercellular CO_2_ was significantly lower in VC than VV and control (Figure 7) which illustrated the silencing of CtPPO increased the utilization of carbon resource. These results help to increase understanding that CtPPO might influence the carbon fixation by regulating NTRC system.

### 3.3. Silencing of PPO Enhanced the glycolysis in C. terniflora

Glycolysis is a central metabolic pathway in plants that oxidizes hexoses to provide ATP, reduces power and pyruvate, and produces precursors for anabolism [47]. Studies have proved that the increase of PGK, and BPGM in *Arabidopsis thaliana* provide energy supply by ATP production [48,49]. Arabidopsis double mutants lacking BPGM enzyme activity exhibited defects in blue light, low CO_2_, and abscisic acid-regulated stomatal movements [49], which are responsible for all gaseous diffusion and can control photosynthetic CO_2_ uptake to influence photosynthesis [50]. The increased glycolytic proteins in root of oat enhanced ATP production and promoted adaptation to anaerobic conditions [51]. It has been suggested that the stomatal red-light response signal may be provided by the redox state of photosynthetic electron transport chain components, such as the redox state of plastoquinone and production of ATP [52]

In our study, the increases in GPUT, FBA, GAPDH, PGK, Enolase, and PEPC in CtPPO-silenced plants demonstrated the activation of glycolysis, which could provide more ATP for energy metabolism in *C. terniflora*. Further analysis also proved the enhanced stomatal conductance by the silencing of CtPPO in VC (Figure 7). The interaction of glyceraldehyde-3-phosphate dehydrogenase and phospholipase D might provide a direct connection between signal transduction, energy metabolism, and growth control in plant response to stress conditions [53]. Yasmeen’s study suggest that Cu nanoparticles might enhance the tolerance of wheat to drought and salinity by increasing the glycolysis related protein abundance [54]. These results indicate the decrease in CtPPO activity in *C. terniflora* might elevate its stress tolerance by activating glycolysis metabolism.

### 3.4. Artificial Interference with PPO Activity Has Potential Applications in Agricultural Production

PPOs catalyze the oxidation of phenols to quinones that subsequently react with amino acids or proteins to form brown and black pigments, greatly reducing the appearance of quality of wheat products [55]. The role of plant PPO in postharvest browning has been the primary focus of research [56]. Although there is no evidence that high PPO activity is associated with depressed nutritional value, the darkened color still negatively affects consumer choice [57]. Studies on wheat concluded that the development of wheat cultivars with low grain PPO activity is one of the main objectives in wheat breeding programs [58]. Photosynthesis is the major trait for any further increase in the yield potential of crops [59]. Various genetic engineering approaches for enhancing C3 plant photosynthesis have been consistently proposed over the past years to improve the crop productivity [60,61,62]. Our study clearly suggests the potential for suppression of PPO activity on photosynthesis in *C. terniflora*, which indicates the feasibility of artificially mediating photosynthesis through the control of PPO gene expression. Nevertheless, it is undeniable that PPO has positive effects on plant defense in response to abiotic or biotic stresses as many PPO genes have been shown to be upregulated by wounding, pathogens, and hormones [63]. However, latent PPO enzymes can be activated by interactions with their substrates [64], occurring in great measure when plants are under stress. All in all, our current work provides a prospective approach for increasing crop yield in agricultural production.

## 4. Material and Methods

### 4.1. Plant Materials and Growth Conditions

*C. terniflora* DC. seeds sprouted in incubators and the sprouts were sown into seedbeds. The seedlings were then transplanted into pots and placed in a greenhouse, which was controlled at 28–30 °C, 70–80% relative humidity, and 160 μmol m^−2^ s^−1^ of white light irradiance. In the garden, conditions ranging from the soil to microclimate were equivalent among all plant samples. After six weeks, the plants were used for experiments [28].

### 4.2. High Level UVB and Dark Treatment

For HUV-B+D, 6-week-old plants were exposed to 104.4 kJ m^−2^ d^−1^ of UV-B irradiation at conditions of 25–30 °C and 80% relative humidity in a cabinet. Plants before irradiated were regarded as starting point samples. Intensity of UV-B irradiation was determined by a UV Light Meter (Beijing Normal University, Beijing, China). After irradiated by HUV-B for 5 h, plants were incubated in dark for 48 h. Leaves in the basal 10 to 60 cm of each experimental plant were collected as simples for further proteomic analyses. Five leaves were collected for each replicate and 3 independent biological replicates were assessed [28].

### 4.3. RNA Extraction and Cloning of CtPPO Gene

An RNeasy Plant Mini kit (Qiagen, Hilden, Germany) was used to obtain total RNA. Quantity and quality of obtained RNA were determined using an Agilent 2100 Bioanalyzer (Agilent Technologies, Palo Alto, CA, USA). The extracted total RNA was used as template to synthesize first-strand complementary DNA (cDNA) using an Oligo (dT) and OneScript^TM^ Reverse Transcriptase OneScrpt^TM^ cDNA Synthesis Kit (Applied Biological Materials Inc., Vancouver, BC, Canada). The first-strand cDNA was utilized as template for PCR using the primers listed in Table S5, which was designed based on the single EST sequence of a suspected CtPPO gene in the transcriptome of *C. terniflora* and amplification was performed as described [28]. The amplified PCR fragments, which were approximately 264 bp in length, were isolated, inserted into the pMD^TM^18-T Vector (pMD^TM^18-T Vector Cloning Kit, Takara, Kyoto, Japan), and sequenced. The complete coding sequence of CtPPO was obtained using 5′- and 3′-rapid amplification of cDNA ends (RACE) with internal specific primers (Table S3) using the SMARTer RACE cDNA Amplification Kit (Takara) according to the manufacturer’s instructions.

The downstream sequence of CtPPO was obtained using a PCR-based genome walking approach with the Genome Walking Kit (Takara). Three designed primers (gene specific primer (GSP); Table S2) and 4 internal short degenerate arbitrarily primed (AP) primers included in the kit (AP1, AP2, AP3, and AP4) were used to perform genome-walking PCR. The PCR conditions and designed primer Tm were based on the manufacturer’s instructions. Three-step nested PCR was performed to increase specificity. The nested PCR products were excised, purified, and sequenced.

### 4.4. Construction of CtPPO Virus-Derived Vectors

A 439-bp fragment of the obtained CtPPO gene was amplified using Permix TaqTM (Ex TaqTM Version 2.0 plus dry; Takara) and inserted into a pMD^TM^18-T vector (pMD^TM^18-T Vector Cloning Kit, Takara) using primers TXPPO-F and TXPPO-R (Table S4) containing XbaI and BamHI restriction enzyme sites. The CtPPO sequence was harvested from the pMD^TM^18-T vector using the EcoRI restriction enzyme and inserted into the EcoRI site between the CaMV 35S promoter (2 × 35S) and NOS terminator of the pTRV2 vector. The orientation of the plasmid with pTRV2-CtPPO (Figure S1) was verified by sequencing. Assembly of the pTRV vector was performed as described [65]. The plasmids were sequenced to verify correct insertion of the fragment and transformed into *Agrobacterium tumefaciens* GV3101.

### 4.5. Virus-Induced Gene Silencing

VIGS was performed according to the method described by Salim et al. [66]. The GV3101 strains of *A. tumefaciens* carrying pTRV1, pTRV2, and pTRV2-PPO were stored at −80 °C. Agrobacteria were cultured in 300 mL of Luria-Bertani liquid medium containing 10 mM 2-(N-morpholino) ethanesulfonic acid (MES), 50 μg mL^−1^ kanamycin, and 20 μM acetosyringone. Then the cultures were centrifuged at the speed of 5000× *g* for 10 min and the resulting bacterial pellets were resuspended in 5 mL infiltration buffer containing 10 mM MgCl_2_, 10 mM MES and 200 μM acetosyringone, and incubated at 28 °C with shaking for 3 h. The mixed suspensions were used for surface infiltration of three-week-old *Nicotiana benthamiana* seedlings using a needleless syringe and 4–6 leaves were infected. The PDS (phytoene desaturase) gene was used as a marker for the evaluation of the efficiency of VIGS in CtPPO genes silencing in *N. benthamiana*. The VIGS *N. benthamiana* plants were grown in a greenhouse and the TRV virus was detected by PCR to confirm infection of the *N. benthamiana* leaves. The successfully infected *N. benthamiana* leaves were used for the abrasion inoculation of *C. terniflora* leaves as described below.

Virus-infected *N. benthamiana* leaves (1 g) were ground with 2 mL of 10 mM potassium phosphate buffer (pH 7) containing 1–2% *w/v* Celite 545 AW abrasive (Sigma-Aldrich, Milwaukee, WI, USA) using a mortar and pestle. The resulting homogenate was used for inoculation by the abrasion method on all tender leaves of 3-week-old *C. terniflora* plants. Two groups of plants, one containing the pTRV virus vector and the other the pTRV-PPO virus vector, were prepared and each was inoculated, where six plants were used as biological replicates.

### 4.6. Protein Extraction, Enrichment, and Digestion for Proteomics Analysis

The tissues were ground in liquid nitrogen. For protein extraction, the phenol extraction method was performed according to a previously published protocol [67]. Briefly, 6 g of frozen powder was suspended in 15 mL of homogenization medium containing 50% (*v*/*v*) phenol, 100 mM KCl, 50 mM EDTA, 0.2% (*v*/*v*) 2-mercaptoethanol, 1 mM PMSF, 700 mM sucrose, and 500 mM Tris-HCl at pH 7.5, shaken at 37 °C for 15 min, and then centrifuged at the speed of 2500× *g* at 4 °C for 20 min. Four volumes of precipitation solution (0.1 M ammonium acetate in 100% methanol) were added in to the phenolic phase collected to precipitate the proteins. The precipitate was collected by centrifugation for 10 min at the speed of 2500× *g*. The supernatant was discarded and washed with precipitation solution. Precipitation was allowed to occur at −20 °C for 3 h and the precipitate was obtained by centrifugation, which was repeated twice. The collected proteins were dissolved in 0.5 mL of 50 mM ammonium bicarbonate (ABC). Protein concentrations were determined using the Bradford assay with bovine serum albumin as the standard.

The protein (100 μg) and an additional 50 mM ABC were transferred into a new tube to create a final volume of 100 μL. The protein was reduced with 10 mM dithiothreitol for 1 h at 55 °C and alkylated with 50 mM iodoacetamide at 37 °C for 30 min in darkness, then digested with sequence-grade modified trypsin (Promega, Fitchburg, WI, USA). Trypsin and 1% lysyl endopeptidase were added to the protein and then the solutions were incubated in a water bath at 37 °C for 4 h and another 1% enzyme was added. The solutions were incubated at 37 °C for 12 h, centrifuged at 10,000× *g*, and then dried in a vacuum. Trypsinized peptides in 200 µL of 0.1% trifluoroacetic acid (TFA) and 0.5% acetonitrile were loaded onto activated and balanced C18 SPE columns (Sep-Pak C18; Waters, Milford, MA, USA). After washing the column with 200 µL of 0.1% TFA and 0.5% acetonitrile, 300 µL of 0.1% TFA and 60 % acetonitrile were added and then the elution solution was collected and dried in a vacuum. Obtained supernatant was collected for subsequent analysis.

### 4.7. Nano-HPLC-MS/MS Analysis

The peptides were resuspended in 100 µL of solvent A (water with 0.1% formic acid; B: ACN with 0.1% formic acid), separated by nano-UPLC, and analyzed by online electrospray tandem MS, which were performed on a Nano Aquity UPLC system (Waters Corporation, Milford, MA, USA) connected to a quadrupole-Orbitrap mass spectrometer (Q-Exactive; Thermo Fisher Scientific, Bremen, Germany) equipped with an online nano-electrospray ion source. Peptide samples (2 µL) were loaded onto the trap column (Thermo Scientific Acclaim PepMap C18, 100 μm × 2 cm) with a flow of 10 μL/min for 3 min and subsequently separated on an analytical column (Acclaim PepMap C18, 75 μm × 25 cm) with a linear gradient of 1 to 30% solvent B (acetonitrile with 0.1% formic acid) in 95 min. The column was re-equilibrated at the initial conditions for 15 min. The column flow rate was maintained at 300 nL/min. The electrospray voltage of 2.0 kV versus the inlet of the mass spectrometer was used.

The Q-Exactive mass spectrometer was set at the data-dependent mode allowing the machine switch between MS and MS/MS acquisition automatically. Survey full-scan MS spectra (*m*/*z* 350–1600) were acquired with a mass resolution of 70 K followed by fifteen sequential high-energy collisional dissociation MS/MS scans with a resolution of 17.5 K. One microscan was recorded using a dynamic exclusion of 30 s in all cases. The MS/MS fixed first mass was set at 100.

### 4.8. Protein Identification Based on Mass Spectrometry Data

Tandem mass spectra were extracted by Proteome Discoverer software (Thermo Fisher Scientific, version 1.4.0.288, Bremen, Germany). Charge state deconvolution and deisotoping were not performed. All MS/MS samples were analyzed using Mascot (Matrix Science, London, UK; version 2.3). Mascot was set up to search the Uniprot database (Taxonomy: Viridiplantae, 5716577 entries, https://www.uniprot.org/) assuming the digestion enzyme trypsin. Mascot was searched with a fragment ion mass tolerance of 0.050 Da and a parent ion tolerance of 10.0 PPM. Carbamidomethyl of cysteine was specified in Mascot as fixed modifications. Oxidation of methionine was specified in Mascot as a variable modification. Minimum peptide length was set to six amino acids the false discovery rate was set to 0.01 for peptide identifications. Identified peptides shared between two proteins were combined and reported as one protein group. The minimum requirement for the identification of a protein was a minimum of 2 matched peptides and a *p*-value < 0.05. Spectral counting was used to estimate relative protein abundance [68]. The mass spectrometry proteomics data have been deposited to the ProteomeXchange Consortium via the PRIDE [69] partner repository with the dataset identifier PXD011439.

### 4.9. Functional Annotation

Protein functions were categorized using MapMan bin codes (available online: http://mapman.gabipd.org/) [70]. Annotations were transferred to the *Arabidopsis* genome with consideration for orthologous genes to predict functions of identified proteins from *C. terniflora*. Pathway mapping of identified proteins was performed using the Kyoto Encyclopedia of Genes and Genomes (KEGG) database (available online: http://www.genome.jp/kegg/) [71].

### 4.10. Cluster Analysis of Protein Abundance

Protein abundance ratios were used for cluster analysis, performed on Cluster 3.0 software version 3.0 (available online: http://bonsai.hgc.jp/~mdehoon/software/cluster/) [72].

### 4.11. Phylogenetic Analysis

The amino acid sequences of CtPPO were aligned using CLUSTALW (available online: http://www.clustal.org/) [73] and a neighbor-joining phylogenetic tree was computed with MEGA6 (available online: http://www.clustal.org/) using 1000 bootstrapped replicates [74].

### 4.12. qRT-PCR

Total RNA of leaves was extracted using an RNA isolation kit (Huayueyang, Beijing, China) and served as the template for reverse transcription using 5× All-In-One RT MasterMix with an AccuRT Genomic DNA Removal Kit (Applied Biological Materials, Richmond, BC, Canada) to obtain cDNA. Next, qRT-PCR was performed on a Bio-Rad IQ2 Multicolor Real-Time PCR Detection System (Bio-Rad, Hercules, CA, USA) with EvaGreen 2× qPCR Master Mix-iCycler (Applied Biological Materials) as the fluorescent dye. GAPDH, a housekeeping gene, was used as a standard for relative quantification of target genes. The gene-specific primers are listed in Additional file 1: Table S1. Three biological replicates were assessed and the relative expression levels were calculated using the 2^−ΔΔ*C*t^ method [26].

### 4.13. PPO Enzymatic Activity

Enzymatic activity of PPO was assayed as described by Marko et al. [75]. Frozen leaf (0.5 g) was homogenized with 1 mL of 50 mM sodium phosphate buffer (pH 7.5) containing 1% (*w*/*v*) polyvinyl polypyrrolidone and 0.1 mM EDTA using a pre-chilled mortar and pestle. After extraction in an end-over-end shaker at 4 °C for 20 mins, the mixture was centrifuged at 16000× *g* for 20 min at 4 °C and the resulting supernatant was used in enzyme extraction.

Then, 800 μL of 0.1 M sodium phosphate buffer (pH 6.0) containing 50 mM (+)-catechin (Carl Roth, Karlsruhe, Germany) was mixed with 150 μL of enzyme extraction sample and incubated for 15 min at 37 °C. The reaction was terminated by adding 150 μL of 6 N HCl and the absorbance at 420 nm was recorded and used in further calculations. Protein concentration was determined according to Bradford using bovine serum albumin as the reference protein. Specific PPO activity was calculated as units (U) per mg sample protein with 1 U corresponding to an absorbance change of 0.01/min. Since levels of PPO activity in the control plants were somewhat variable between independent experiments, data were normalized to the specific PPO activity in leaves of healthy wild-type controls and the results are shown as fold induction (with 1 corresponding to a specific activity of 3.2 to 23 U/mg).

### 4.14. Analysis of Photosynthesis Characteristics

An Li-6400 Portable Photosynthesis System (Li-Cor, Lincoln, NE, USA) equipped with a red/blue LED light source were used to measure the photosynthesis characteristics of the leaves, and was operated using air from a large volume with a stable CO_2_ partial pressure. All measurements were carried out at a photon flux density of 500 μmol m^−2^ s^−1^ at 28 °C and made after stable reading was achieved. The photosynthesis rate (μmol m^−2^ s^−1^), stomatal conductance (mol m^−2^ s^−1^), and transpiration rate (mmol m^−2^ s^−1^) of each leaf were recorded. Each experiment was repeated for different plants three times and three independent biological replicates were assessed.

### 4.15. Analysis of Chlorophyll Content

*C. terniflora* leaves (0.2 g) were cut up and blended with 10 mL of 95% alcohol in a tube. The tube was covered and the leaves soaked in the dark until they had turned completely white. The absorbances of the extracting solutions were measured at wavelengths of 665, 649, and 470 nm separately. The chlorophyll content was calculated using Chlorophyll a (Ca) = 13.95 D_665_ − 6.88 D_649_, Chlorophyll b (Cb) = 24.96 D_649_ − 7.32 D_665_, Carotenoid = (1000 D_470_ − 2.05 Ca − 114 Cb)/245, and Total chlorophyll = Ca + Cb.

### 4.16. Statistical Analysis

Statistical significance comparing two groups was determined using Student’s *t*-test and multiple groups using one-way ANOVA followed by Tukey’s test. Statistical evaluation was performed on SPSS statistical software version 22.0 (IBM, Armonk, NY, USA). A *p*-value <0.05 was considered statistically significant.

### 4.17. Accession Codes

The nucleotide sequence of CtPPO has been deposited in the GenBank™ database under the accession number MK070494.

## 5. Conclusions

PPO is an oxidoreductase that plays a crucial role in the biosynthesis of secondary metabolites. *C. terniflora* is a Chinese folk medical resource with potential pharmaceutical value for the treatment of various diseases [21]. High-intensity UV-B and dark incubation (HUV-B+D), which are helpful stressors for medicinal plants [28]. Omics technologies were used on *C. terniflora* to prospectively understand the inherent mechanism underlying its medicinal quality [24,26]. To clarify the in vivo physiological functions of PPO, we performed a comparative proteomic analysis on the leaves of *C. terniflora* with down-regulated PPO activity by VIGS. The main findings are as follows: (i) The differentially expressed proteins were mainly functioned in photosynthesis, glycolysis and redox in the VC plants; (ii) the differentially expressed proteins related to photosynthesis were mainly involved in light reaction and Calvin cycle; (iii) the expression level of adenosine triphosphate (ATP) synthase, the content of chlorophyll, and the photosynthesis rate were increased in VC plants compared to VV plants pre- and post HUV-B+D. Taken together, these results indicate that the silence of PPO can activate the glycolysis process, regulate Calvin cycle and provide ATP for energy metabolism to elevate the plant photosynthesis. And this study provides a prospective approach for increasing crop yield in agricultural production.

## Figures and Tables

**Table 1:** Differential Expressed Proteins in *Clematis terniflora* DC. between VV and VC after HUV-B+D treatment (1.55 > fold change > 1.27).

No.	Protein ID ^a^	Description	Abundance		FC	*p*-Value	Annotation ^b^
			VV	VC			
	Increased						
1	F6HCT7	Uncharacterized protein	0.01	0.02	1.55	0.01	stress
2	M5W912	Ferredoxin-NADP reductase	0.04	0.06	1.51	0.00	PS
3	A0A2G5CER3	Uncharacterized protein (Fragment)	0.01	0.01	1.50	0.03	signaling
4	A0A200RAD1	UTP-glucose-1-phosphate uridylyltransferase	0.02	0.03	1.48	0.03	glycolysis
5	I6P9I5	Cytosolic ascorbate peroxidase (Fragment)	0.03	0.04	1.47	0.02	redox
6	J3MAL0	Ferredoxin-NADP reductase	0.04	0.06	1.47	0.00	PS
7	M4CGU3	Uncharacterized protein	0.02	0.02	1.45	0.02	PS
8	Q42908	2,3-bisphosphoglycerate-independent phosphoglycerate mutase	0.01	0.02	1.42	0.04	glycolysis
9	A0A1J3H2G2	Ubiquitin-NEDD8-like protein RUB1 (Fragment)	0.27	0.38	1.40	0.01	protein
10	A0A2G5F096	Uncharacterized protein	0.02	0.03	1.40	0.05	development
11	C5YTC0	Uncharacterized protein	0.02	0.03	1.39	0.00	not assigned
12	A0A067L6G5	Uncharacterized protein	0.02	0.03	1.39	0.00	amino acid metabolism
13	W9RXI1	Glycerate dehydrogenase	0.08	0.11	1.38	0.00	PS
14	Q1EP00	Chlorophyll a-b binding protein, chloroplastic	0.05	0.07	1.37	0.03	PS
15	W9SCQ6	Ferredoxin-NADP reductase	0.05	0.07	1.36	0.00	PS
16	A0A2G5DAJ3	Carbonic anhydrase	0.03	0.04	1.36	0.04	TCA
17	W8TP69	Glycerate dehydrogenase-like protein	0.04	0.06	1.36	0.02	PS
18	A0A0K9P513	Phosphoglycerate kinase	0.08	0.10	1.35	0.01	PS
19	A0A0K9Q3W1	70 kDa heat shock protein	0.06	0.07	1.35	0.01	stress
20	A0A1J6I7J0	2-cys peroxiredoxin bas1, chloroplastic	0.05	0.07	1.35	0.01	redox
21	C0PRV0	Lactoylglutathione lyase	0.03	0.04	1.33	0.02	Biodegradation of Xenobiotics
22	Q19U04	NADH-dependent hydroxypyruvate reductase (Fragment)	0.11	0.15	1.33	0.00	PS
23	D2XUU3	Chloroplast managanese stabilizing protein (Fragment)	0.17	0.22	1.32	0.04	PS
24	A0A0A0KBL8	Uncharacterized protein	0.03	0.04	1.32	0.00	PS
25	A5BVF4	Uncharacterized protein	0.16	0.21	1.32	0.02	PS
26	A0A200QG47	Aminotransferase	0.05	0.06	1.32	0.02	PS
27	W1P8B5	Uncharacterized protein	0.01	0.01	1.31	0.05	OPP
28	A0A0D3B1C7	Uncharacterized protein	0.02	0.02	1.30	0.02	OPP
29	K7KB09	Uncharacterized protein	0.03	0.04	1.30	0.01	hormone metabolism
30	K4BW79	2-methylene-furan-3-one reductase	0.06	0.07	1.30	0.00	misc
31	A0A2H5NQP8	Uncharacterized protein	0.08	0.10	1.30	0.01	PS
32	A0A0D2Q3K9	Uncharacterized protein	0.04	0.05	1.30	0.00	stress
33	A0A200PYZ1	ATPase	0.08	0.11	1.29	0.01	PS
34	A1BQW9	Transketolase (Fragment)	0.05	0.06	1.29	0.00	PS
35	A0A251VGE5	Putative photosystem I PsaA/PsaB	0.05	0.06	1.29	0.03	PS
36	A0A1D8H339	2-Cys peroxiredoxin	0.07	0.09	1.28	0.01	redox
37	S8EAM3	Heat shock protein hsp70 (Fragment)	0.03	0.03	1.28	0.00	stress

^a^ Protein ID, according to UniProtKB/Swiss-Prot database. ^b^ Function, protein function categorized using MapMan bin codes. FC, fold change; PS, photosynthesis; TCA, tricarboxylic acid; OPP, oxidative pentose phosphate; misc: miscellaneous.

**Table 2:** Differential Expressed Proteins in *Clematis terniflora* DC. between VV and VC at starting point (fold change > 1.40).

No.	Protein ID ^a^	Description	Abundance		FC	*p*-Value	Annotation ^b^
			VV	VC			
	Increased						
1	A0A151U9E4	Uncharacterized protein	0.02	0.03	1.92	0.01	redox
2	Q8M9K2	Ribulose-bisphosphate carboxylase (Fragment)	0.79	1.46	1.84	0.00	PS
3	K8ECB3	Thioredoxin	0.04	0.08	1.82	0.00	redox
4	A0A067KU12	UTP-glucose-1-phosphate uridylyltransferase	0.03	0.04	1.57	0.02	glycolysis
5	A0A068TPY5	Uncharacterized protein	0.03	0.05	1.53	0.01	PS
6	A0A2G3DEA2	3-oxo-Delta(4,5)-steroid 5-beta-reductase	0.03	0.04	1.45	0.02	development
7	A1X444	Ribulose-1,5-bisphosphate carboxylase/oxygenase large	0.27	0.39	1.44	0.00	PS
8	A0A1U8LIR6	2-methyl-6-phytyl-1,4-hydroquinone methyltransferase	0.02	0.02	1.43	0.01	secondary metabolism
9	A0A200RAD1	UTP-glucose-1-phosphate uridylyltransferase	0.03	0.04	1.42	0.04	glycolysis
10	A0A2G5DW13	Uncharacterized protein	0.04	0.06	1.42	0.01	redox
11	A0A067KC46	Ribulose bisphosphate carboxylase small chain	0.06	0.09	1.41	0.02	PS
12	W9QII5	Peroxiredoxin Q	0.03	0.04	1.41	0.02	redox
13	A0A061EH79	Ribulose bisphosphate carboxylase small chain	0.05	0.07	1.41	0.00	PS

^a^ Protein ID, according to UniProtKB/Swiss-Prot database. ^b^ Function, protein function categorized using MapMan bin codes. FC, fold change; PS, photosynthesis.

## Supplementary Materials

The following are available online at http://www.mdpi.com/1422-0067/19/12/3897/s1.

## Abbreviations

3-PGA	3-Phosphoglycerate
ABA	Abscisic acid
ABC	Ammonium bicarbonate
ACN	Acetonitrile
ATP	Adenosine triphosphate
BPGM	Phosphoglycerate mutase
*C. terniflora*	*Clematis terniflora*
CtPPO	PPO gene in *C. terniflora*
DEPs	Differentially expressed proteins
ETC	Electron transport chain
FBA	Fructose-1,6-bisphosphate aldolase
FBPase	Fructose-1,6-bisphosphatase
Fd	Ferredoxin
FTR	Fd-TRX reductase
GAP	Glyceraldehyde 3-phosphate
GAPDH	Glyceraldehyde-3-phosphate dehydrogenase
GPI	Glucose-6-phosphate isomerase
GPUT	UTP-glucose-1-phosphate uridylytransferase
HCD	High energy collisional dissociation
HUV-B+D	High level of UV-B and dark
KEGG	Kyoto Encyclopedia of Genes and Genomes
MES	2-(N-morpholino) ethanesulfonic acid
NTRC	NADPH-dependent TRX reductase
ORF	Open reading frame
PDS	Phytoene desaturase
PEP	Phosphoenolpyruvate
PEPC	Phosphoenolpyruvate carboxylase
PGK	Phosphoglycerate kinase
PPO	Polyphenol oxidase
PRK	Phosphoribulokinase
PS I	Photosystem I
PS II	Photosystem II
PS	Photosystem
RACE	5′ Rapid amplification of cDNA ends
Rubisco	Ribulose-1,5-bisphosphate carboxylase/oxygenase
RuBP	Ribulose 1,5-bisphosphate
SBPase	Sedoheptulose-1,7-bisphosphatase
TCA	Tricarboxylic acid
TFA	Trifluoroacetic acid
TK	Transketolase
TRV	Tobacco rattle virus
TRX	Thioredoxin
UTR	Untranslated Region
VIGS	Virus-induced gene silencing
VC	VIGS-CtPPO
VV	VIGS-vector
