# Supplementary material for: Comparative Proteomic Analysis Reveals Elevated Capacity for Photosynthesis in Polyphenol Oxidase Expression-Silenced Clematis terniflora DC. Leaves
Source: Int J Mol Sci. 2018 Dec 5;19(12):3897. doi: 10.3390/ijms19123897 (PMC6321541; doi:10.3390/ijms19123897)
Supplement: Supplementary file 1 [file ijms-19-03897-s001.zip › ijms-368388 sp for final/Supplementary materials/Supplemental Figures.pdf]

Supplement Figure 1

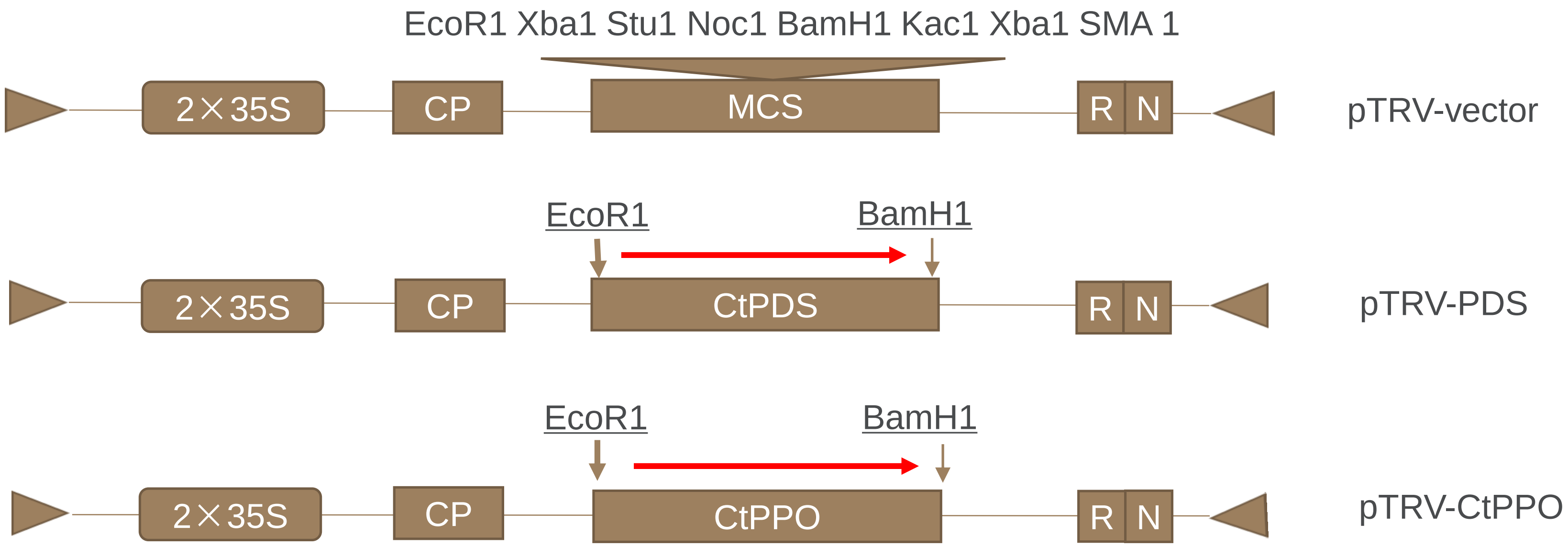

Construct virus-derived vectors: pTRV-vector, pTRV-PDS, and pTRV-CtPPO

Supplement Figure 2

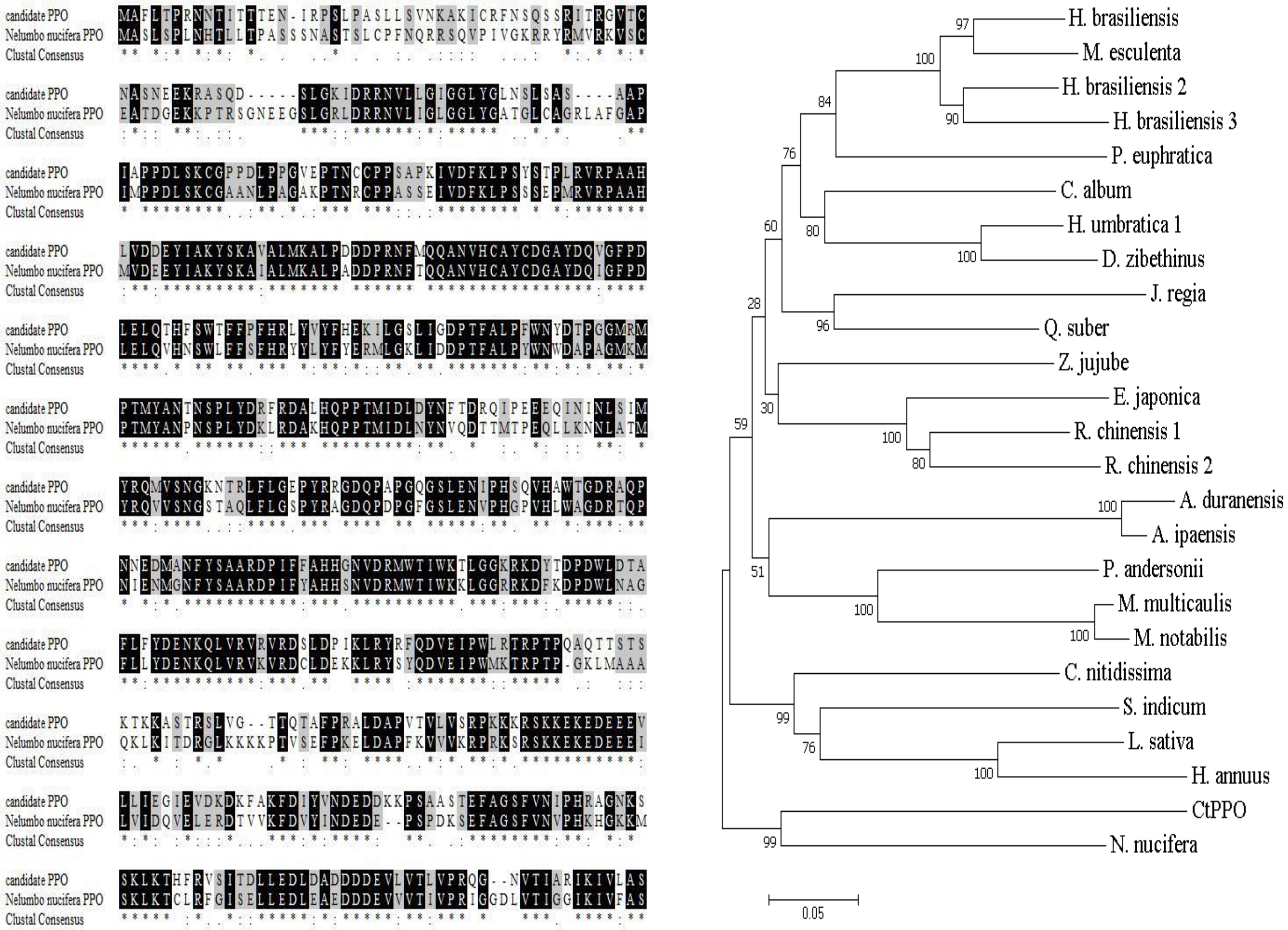

(a) (b)

(a) Multiple sequence alignments of the deduced amino acid sequences of CtPPO and *Nelumbo nucifera* PPO. Black background, completely conserved region; grey back ground, partly conserved region; (b) Phylogenetic relationshis between CtPPO and *N. nucifera* (ADC92563.1), *Morus alba var. multicaulis* (ALF12286.1), *Ziziphus jujube* (NP_001310781.1), *Eriobotrya japonica* (AFO55217.1), *Camellia nitidissima* (ACM43505.1), *Herrania umbratica* (XP_021295073.1), *Hevea brasiliensis 1* (XP_021650904.1), *Morus notabilis* (XP_010100570.1), *Hevea brasiliensis 2* (XP_021662146.1), *Manihot esculenta* (XP_021599845.1), *Lactuca sativa* (XP_023763394.1), *Rosa chinensis 1* (XP_024156865.1), *Juglans regia* (ACN86310.1), *Helianthus annuus* (XP_022020421.1), *Hevea brasiliensis 3* (XP_021649207.1), *Rosa chinensis 2* (XP_024161280.1), *Parasponia andersonii* (PON50737.1), *Quercus suber* (XP_023923556.1), *Arachis duranensis* (XP_020988465.1), *Arachis ipaensis* (XP_016182086.1), *Canarium album* (AEY78528.1), *Populus euphratica* (AEL95440.1), *Durio zibethinus* (XP_022720500.1), and *Sesamum indicum* (XP_011096441.1) based on amino acid sequences.

Supplement Figure 3

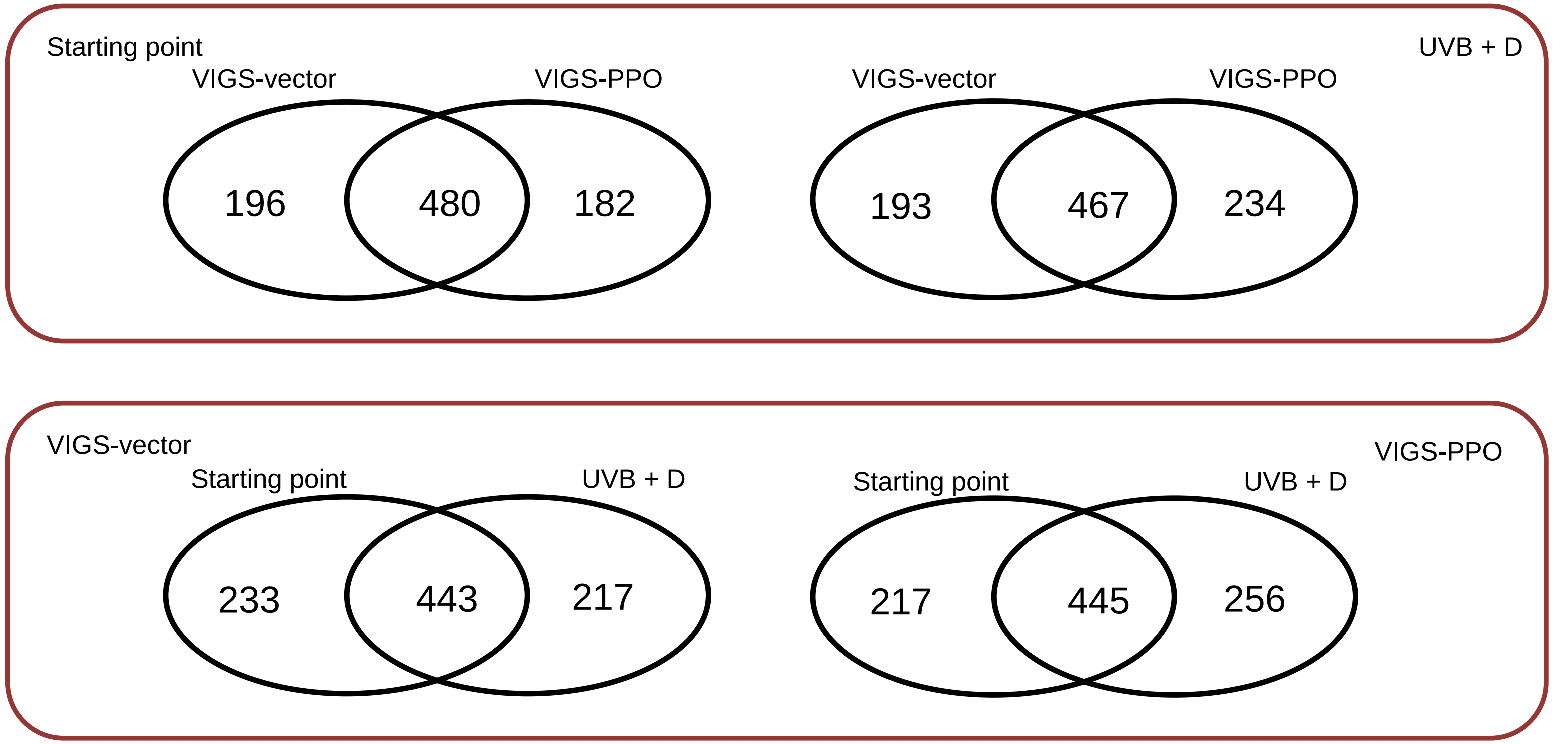

Venn diagrams of differentially changed proteins in *Clematis terniflora* DC. with VIGS-vector and VIGS-CtPPO at starting point and after HUV-B+D treatment

Supplement Figure 4

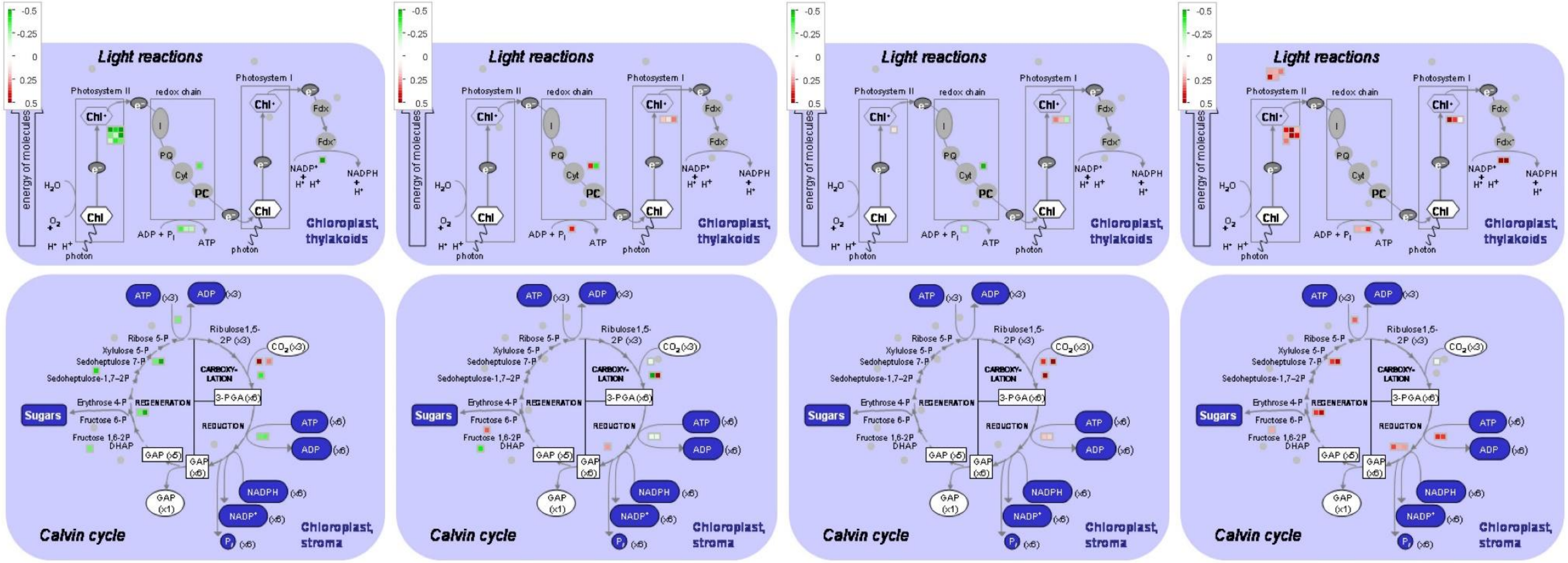

a. VV at HUV-B+D vs starting point

b. VC at HUV-B+D vs starting point

c. VC vs VV at starting point

d. VC vs VV after HUV-B+D

Metabolic pathways of proteins identified in VIGS-vector and VIGS-CtPPO *C. terniflora* DC. Leaves were collected at the starting point and after HUV-B+D treatment. The proteins were grouped into functional categories related to primary metabolism and changes in abundance were visualized using MapMan software. Each square and color indicates the fold-change of a differentially expressed protein. Green and red indicate a decrease and increase, respectively, in fold change compared with the corresponding group: (a) comparison of proteins in VIGS-vector leaves after HUV-B+D treatment at starting point, (b) comparison of proteins in VIGS-CtPPO leaves after HUV-B+D treatment at starting point, (c) comparison of proteins at starting point in VIGS-CtPPO and VIGS-vector plants, and (d) comparison of proteins after HUV-B+D treatment in VIGS-CtPPO and VIGS-vector plants. Abbreviations: OPP, oxidative pentose phosphate; CHO, carbohydrate; TCA, tricarboxylic acid cycle.

Supplement Figure 5

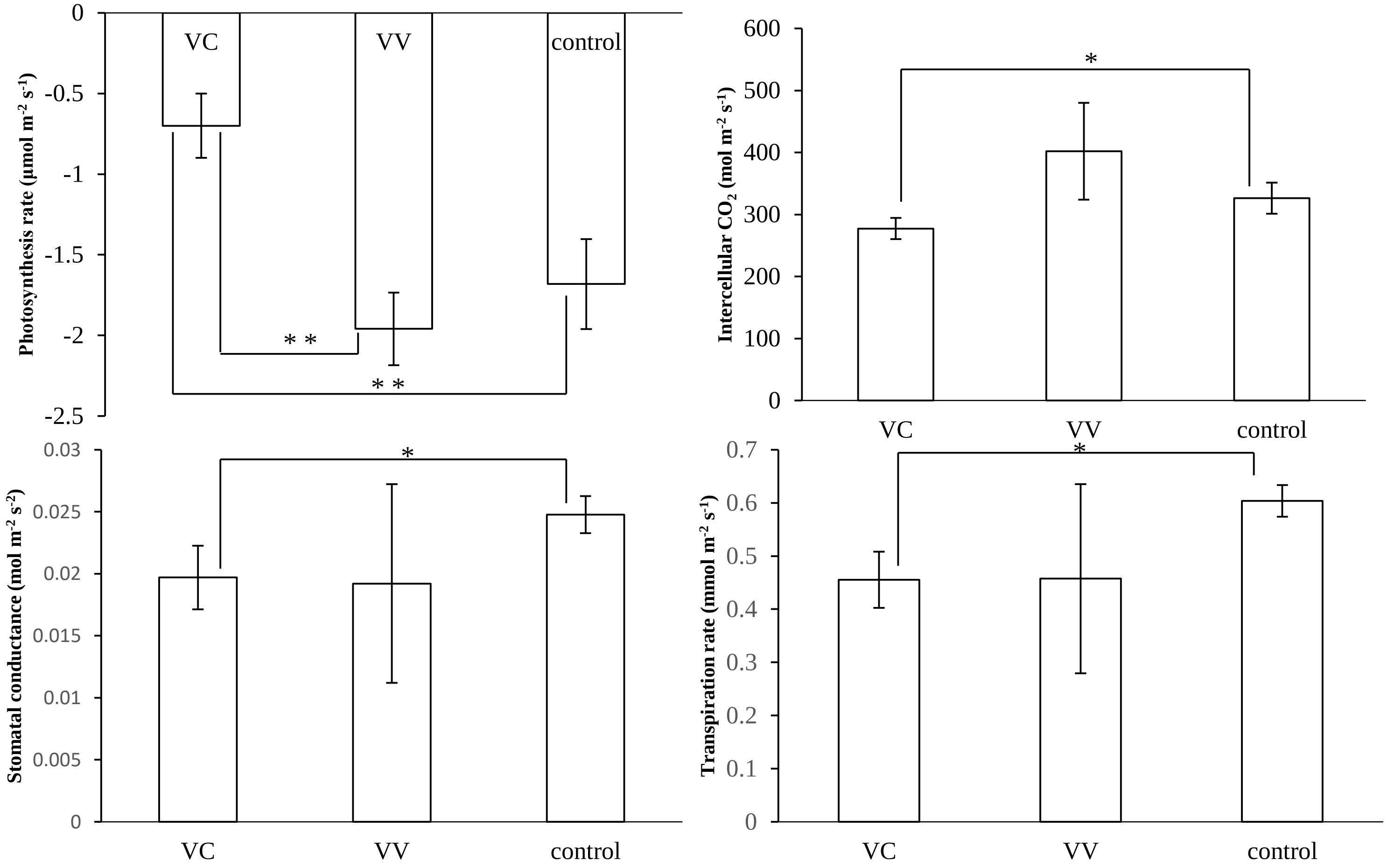

Analysis of photosynthesis characteristics in control, VIGS-vector, and VIGS-CtPPO *C. terniflora* DC. leaves. C. terniflora leaves after HUV-B+D treatment. The photosynthesis rate, intercellular CO2, stomatal conductance, and transpiration rate were measured using an open gas-exchange system. Data are shown as mean ± SD of independent biological replicates. Asterisks indicate significant changes as measured by Student's t-test (*p <0.05, **p<0.01, and ***p<0.001).
