# Supplementary material for: Comparative Proteomic Analysis Reveals Elevated Capacity for Photosynthesis in Polyphenol Oxidase Expression-Silenced Clematis terniflora DC. Leaves
Source: Int J Mol Sci. 2018 Dec 5;19(12):3897. doi: 10.3390/ijms19123897 (PMC6321541; doi:10.3390/ijms19123897)
Supplement: Supplementary file 1 [file ijms-19-03897-s001.zip › ijms-368388 sp for final/Supplementary materials/Supplemental Tables.pdf]

Table S1. List of primers used for qRT-PCR experiments

Gene	Forward primer (5' to 3')	Reverse primer (5' to 3')
CtGAPDH	AACCCTGAGGAGATTCCA	CACCACCCTTCAAGTGAGCAG
CtPPO	GGTTGGACACTGCCTTTC	GATGTAGTCTGGGCTTGG
CtATP	GGGGCTCTCGTTTCCTTTCA	TTGCTCAGGCCATTGAGGAG

Table S2. List of primers used for genome walking PCR experiment

Gene	Primer (5' to 3')
GSP1	GCGACCGTGCCCAACCTA
GSP2	CACGGCAATGTTGACCGAATG
GSP3	ATTGGTTGGACACTGCCTTTC

Table S3. List of primers used for RACE experiment

Gene	Primer (5' to 3')
AUAP	GGCCACGCGTCGACTAGTAC
AAP	GCCACGCGTCGACTAGTACGGGGGGGGGG
RC134-R2	AAATTACGTGGGTCATCATCGGGAAGAG
RC134-R1	TGCAGTTCCAAATCAGGAAAACCAACC
RC134-RT1	GGTAAGGCAAAAGTTGGATCACCAATCA

Table S4. List of primers used for VIGS experiment

Gene	Primer (5' to 3')
TXPPO-F	CGGGATCCGACCGGAGGAATGTTTTAC
TXPPO-R	GCTCTAGATCTCATGAAAATATACGTAC
BAMTXPDS-F	CGGGATCCGTTACCATCTAAAAAGGCCATC
XBATXPDS-R	GCTCTAGACAGTGGAAGGAACACTCCATG

Table S5. Primer for EST sequence in C. terniflora

Gene	Forward primer (5' to 3')	Reverse primer (5' to 3')
PPO	CCAGAAGGGTAAGGCAAAAG	GATGATGAGTACATTGCTAA

Table S6. List of Proteins Identified in Leaf of *Clematis terniflora* DC. in VV

No.	Protein ID[a]	Description	VV at starting point abundance	VV after HUV-B+D abundance	Fold changs	P-value	Annotation
1	A0A067JNK4	Ketol-acid reductoisomerase	0.010118044	0.006183249	0.611111133	0.002192132	amino acid metabolism
2	A0A1S2YIV0	Cysteine synthase	0.034252297	0.023391813	0.682926835	0.010059711	amino acid metabolism
3	C0PRV0	Lactoylglutathione lyase	0.035616438	0.02739726	0.769230769	0.006533376	Biodegradation of Xenobiotics
4	A0A1Z5S503	Serine hydroxymethyltransferase	0.065627031	0.050032488	0.762376232	0.005820687	C1-metabolism
5	A0A103XZB1	Serine hydroxymethyltransferase (Fragment)	0.072575466	0.053307643	0.734513275	0.00417138	C2-metabolism
6	A0A2G5D2H5	Phosphoglycerate kinase	0.161716172	0.138613861	0.857142855	0.038514272	glycolysis
7	A0A2I0XF37	Phosphoglycerate kinase, cytosolic	0.133000831	0.10556941	0.793750003	0.007624696	glycolysis
8	A0A2G5DIJ2	Fructose-bisphosphate aldolase	0.086592179	0.077281192	0.892473118	0.034109429	glycolysis
9	A0A068V643	Uncharacterized protein	0.063813814	0.058558559	0.917647063	0.024896172	glycolysis
10	I1MQ89	Uncharacterized protein	0.079400749	0.070411985	0.886792453	0.04365947	glycolysis
11	A0A1B2H9Q1	Enolase 1	0.05800464	0.050270689	0.866666673	0.034109432	glycolysis
12	A0A0B0PZ82	Acyl-coenzyme A oxidase 4, peroxisomal-like protein	0.011415525	0.017503805	1.533333333	0.01613009	lipid metabolism.lipid degradation
13	A5BAY5	Uncharacterized protein	0.021164021	0.014109348	0.666666682	0.01613009	mitochondrial electron transport / ATP synthesis
14	A0A1D1YMH1	Ferredoxin-dependent glutamate synthase, chloroplastic	0.023575236	0.01701517	0.721739141	0.026039032	N-metabolism
15	A0A2G5EWR5	Uncharacterized protein	0.02712161	0.019247594	0.709677409	0.002122883	N-metabolism
16	A0A2G3AEG8	FAM10 family protein	0.015111111	0.011555556	0.764705895	0.047420656	not assigned
17	C5YTC0	Uncharacterized protein	0.027777778	0.019965278	0.71874999	0.015799841	not assigned
18	A0A2G5EPB1	6-phosphogluconate dehydrogenase, decarboxylating	0.028747433	0.044490075	1.547619062	0.004535416	OPP
19	A0A2G5F864	Uncharacterized protein	0.066823899	0.046383648	0.694117649	0.003233251	protein
20	W1NKM9	Uncharacterized protein	0.04628331	0.035530622	0.767676764	0.00096397	protein
21	A0A1J3H2G2	Ubiquitin-NEDD8-like protein RUB1 (Fragment)	0.22875817	0.267973856	1.171428571	0.032677924	protein
22	D7M7T7	Predicted protein	0.050025523	0.056661562	1.132653059	0.014720594	protein
23	A0A0K9PHU6	Elongation factor	0.010089686	0.016442451	1.629629637	0.04907192	protein
24	A0A2H3ZZH5	LOW QUALITY PROTEIN: elongation factor 2-like	0.013444049	0.0209569	1.558823575	0.004597008	protein
25	A0A2H5P4R5	Uncharacterized protein	0.053191489	0.050531915	0.950000008	6.95E-59	protein
26	W1NXE4	Uncharacterized protein	0.010673235	0.015599343	1.461538417	0.0132356	protein
27	A0A2G5DB79	Uncharacterized protein	0.006920415	0.011534025	1.666666714	0.016130078	protein
28	M5XQU6	Uncharacterized protein	0.037216828	0.027508091	0.739130448	0.043659477	protein
29	A0A2G5EYV9	Uncharacterized protein	0.048654244	0.035196688	0.723404261	0.043814454	protein

30	A0A2I0APB2	Fructose-bisphosphate aldolase, chloroplastic	0.061696658	0.049700086	0.805555562	0.017797405	PS
31	F6GWQ0	Fructose-bisphosphate aldolase	0.072390573	0.058080808	0.802325577	0.013158071	PS
32	A0A0K9P513	Phosphoglycerate kinase	0.094941095	0.076230076	0.802919706	0.020676869	PS
33	A0A2H5NQP8	Uncharacterized protein	0.100487439	0.080614923	0.802238805	0.021963587	PS
34	A0A0E0A154	Phosphoglycerate kinase	0.196707819	0.156378601	0.79497908	0.029414263	PS
35	A0A2G5ESQ6	Phosphoribulokinase	0.160130719	0.129084967	0.806122449	0.00077015	PS
36	A5C3D4	Uncharacterized protein	0.087570622	0.072033898	0.82258064	0.037996593	PS
37	V5NC92	Ribulose bisphosphate carboxylase/oxygenase activase 2	0.021483022	0.016632017	0.774193559	0.024896163	PS
38	A0A0A0KBG2	Uncharacterized protein	0.070323488	0.054149086	0.770000003	0.012939919	PS
39	C6YBD7	Chloroplast ribulose-1,5-bisphosphate carboxylase activase (Fragment)	0.061111111	0.044444444	0.727272721	0.01713295	PS
40	Q8M9K2	Ribulose-bisphosphate carboxylase (Fragment)	0.794117647	1.558823529	1.962962964	0.000114752	PS
41	A1X444	Ribulose-1,5-bisphosphate carboxylase/oxygenase large subunit (Fragment)	0.268156425	0.418994414	1.5625	0.000235722	PS
42	F6JSX0	Ribulose bisphosphate carboxylase large chain (Fragment)	0.211509716	0.292227205	1.38162544	0.002055288	PS
43	Q6Y2Y1	Ribulose bisphosphate carboxylase large chain (Fragment)	0.218773097	0.297117516	1.358108106	0.000955532	PS
44	Q33047	Ribulose bisphosphate carboxylase large chain (Fragment)	0.291666666	0.389880953	1.336734696	3.27E-05	PS
45	Q31895	Ribulose bisphosphate carboxylase large chain (Fragment)	0.285612535	0.378917379	1.326683293	0.000378498	PS
46	Q95631	Ribulose bisphosphate carboxylase large chain (Fragment)	0.331201137	0.43923241	1.326180258	0.000433092	PS
47	Q37220	Ribulose bisphosphate carboxylase large chain (Fragment)	0.363161819	0.480238628	1.32238193	0.000738832	PS
48	B1NL21	Ribulose bisphosphate carboxylase large chain (Fragment)	0.310606061	0.405723906	1.306233062	0.000132676	PS
49	Q37279	Ribulose bisphosphate carboxylase large chain (Fragment)	0.303221288	0.395658263	1.304849885	0.001082767	PS
50	Q4VI78	Ribulose bisphosphate carboxylase large chain (Fragment)	0.357562408	0.462555066	1.293634497	0.000276402	PS
51	Q8WL19	Ribulose bisphosphate carboxylase large chain (Fragment)	0.277065527	0.358262108	1.293059124	5.03E-05	PS
52	Q32553	Ribulose bisphosphate carboxylase large chain (Fragment)	0.332628611	0.429175476	1.290254239	0.000804273	PS
53	C1JAT3	Ribulose-1,5-bisphosphate carboxylase/oxygenase large subunit (Fragment)	0.350961539	0.451923077	1.287671232	0.000503477	PS
54	A0A291L2S1	Ribulose bisphosphate carboxylase large chain	0.390499195	0.500805153	1.282474226	0.000479882	PS

		(Fragment)					
55	J9QH20	Ribulose bisphosphate carboxylase large chain (Fragment)	0.340563991	0.432393348	1.269639066	0.00124491	PS
56	A4H1D1	Ribulose bisphosphate carboxylase large chain (Fragment)	0.285493827	0.360339506	1.262162161	0.00032512	PS
57	Q3T5L7	Ribulose bisphosphate carboxylase large chain (Fragment)	0.399283154	0.499641577	1.251346499	0.001169913	PS
58	Q3I534	Ribulose bisphosphate carboxylase large chain (Fragment)	0.380743982	0.469000729	1.231800767	0.001014737	PS
59	D6MYK9	Ribulose bisphosphate carboxylase large chain	0.377777778	0.464583333	1.229779412	0.005676234	PS
60	Q8WJX3	Ribulose bisphosphate carboxylase large chain (Fragment)	0.397003745	0.486142322	1.224528301	0.000549698	PS
61	Q9TN58	Ribulose bisphosphate carboxylase large chain	0.402105263	0.48631579	1.209424084	0.002407883	PS
62	V5K6K1	Ribulose bisphosphate carboxylase large chain (Fragment)	0.39624183	0.47630719	1.202061856	0.007143112	PS
63	A5X4D3	Ribulose bisphosphate carboxylase large chain (Fragment)	0.478978979	0.575075075	1.20062696	0.002865343	PS
64	Q9GGI5	Ribulose bisphosphate carboxylase large chain (Fragment)	0.520494973	0.623356535	1.197622587	0.000671806	PS
65	D6MYK4	Ribulose bisphosphate carboxylase large chain	0.467361111	0.556944444	1.191679049	0.004002576	PS
66	Q7GH77	Ribulose bisphosphate carboxylase large chain (Fragment)	0.479532163	0.569444444	1.1875	0.000633845	PS
67	D6MYJ6	Ribulose bisphosphate carboxylase large chain	0.475	0.563888889	1.187134503	0.006307982	PS
68	B5L2V5	Ribulose bisphosphate carboxylase large chain (Fragment)	0.461599367	0.547901821	1.18696398	0.00036399	PS
69	Q8ME51	Ribulose bisphosphate carboxylase large chain (Fragment)	0.427645788	0.507559395	1.186868688	0.005997305	PS
70	I2E9A4	Ribulose bisphosphate carboxylase large chain (Fragment)	0.555555556	0.659108088	1.186394558	0.006019217	PS
71	F2X4H9	Ribulose bisphosphate carboxylase large chain (Fragment)	0.521348315	0.617228464	1.183908045	0.001444261	PS
72	Q32701	Ribulose bisphosphate carboxylase large chain	0.456140351	0.539649123	1.183076925	0.000527109	PS
73	Q8MFT9	Ribulose bisphosphate carboxylase large chain (Fragment)	0.556581986	0.658198615	1.182572616	0.000827989	PS
74	G8EEM9	Ribulose bisphosphate carboxylase large chain (Fragment)	0.439224459	0.51901566	1.181663837	0.000197701	PS
75	E7DMY2	Ribulose bisphosphate carboxylase large chain (Fragment)	0.507559395	0.599712023	1.181560283	0.00065968	PS
76	Q33032	Ribulose bisphosphate carboxylase large chain (Fragment)	0.497150997	0.586894587	1.18051576	0.001993375	PS

77	S6CMZ7	Ribulose bisphosphate carboxylase large chain (Fragment)	0.489187174	0.577181208	1.179878048	0.001022399	PS
78	A0A249Y5N8	Ribulose bisphosphate carboxylase large chain (Fragment)	0.53125	0.626602564	1.179487179	0.000565003	PS
79	Q5I221	Ribulose bisphosphate carboxylase large chain	0.477894737	0.563508772	1.179148312	0.001881194	PS
80	L0N8B1	Ribulose bisphosphate carboxylase large chain (Fragment)	0.550759393	0.649080735	1.178519593	0.001727144	PS
81	D6MYI7	Ribulose bisphosphate carboxylase large chain	0.502083333	0.589583333	1.174273859	0.012479962	PS
82	R4QE58	Ribulose bisphosphate carboxylase large chain (Fragment)	0.538576779	0.632209738	1.173852572	0.003882082	PS
83	Q6VW40	Ribulose bisphosphate carboxylase large chain (Fragment)	0.536334913	0.629541864	1.173784978	0.00094154	PS
84	Q7YJM3	Ribulose bisphosphate carboxylase large chain (Fragment)	0.51293422	0.599408721	1.168587896	0.00106689	PS
85	V5T6A6	Ribulose bisphosphate carboxylase large chain (Fragment)	0.465465465	0.543543543	1.167741936	0.040248041	PS
86	A0A2H4UGV5	Ribulose-1,5-bisphosphate carboxylase/oxygenase large subunit (Fragment)	0.454545455	0.529933481	1.165853658	0.005612396	PS
87	I6N589	Ribulose bisphosphate carboxylase large chain (Fragment)	0.462271062	0.537728938	1.163232964	0.002051661	PS
88	I2BGM7	Ribulose bisphosphate carboxylase large chain (Fragment)	0.500379651	0.581624905	1.162367223	0.000933722	PS
89	A0A0N7FMB3	Ribulose bisphosphate carboxylase large chain (Fragment)	0.356025039	0.41314554	1.160439561	0.003881202	PS
90	Q8MCW3	Ribulose bisphosphate carboxylase large chain (Fragment)	0.42303433	0.490586932	1.159685863	0.017939976	PS
91	O47280	Ribulose bisphosphate carboxylase large chain (Fragment)	0.46799117	0.542310522	1.158805032	0.003082643	PS
92	C6G1P2	Ribulose bisphosphate carboxylase large chain (Fragment)	0.556515479	0.640028798	1.150064682	0.004235695	PS
93	A0A1L2YYU1	Ribulose bisphosphate carboxylase large chain (Fragment)	0.24251497	0.278443114	1.148148149	0.003308425	PS
94	A0A0F6TFY2	Ribulose bisphosphate carboxylase large chain (Fragment)	0.511685117	0.574415744	1.122596154	0.01441576	PS
95	A0A142I7A5	Ribulose bisphosphate carboxylase large chain (Fragment)	0.339667458	0.380839271	1.121212121	0.047823466	PS
96	Q8WHW2	Ribulose bisphosphate carboxylase large chain (Fragment)	0.169166667	0.188333333	1.113300493	0.028439261	PS
97	K4EPE7	Ribulose bisphosphate carboxylase large chain (Fragment)	0.472927242	0.521996616	1.103756709	0.022818869	PS
98	B6GUV5	Ribulose bisphosphate carboxylase large chain	0.416309013	0.450643777	1.082474227	0.020775716	PS

		(Fragment)					
99	E0D9L7	Ribulose bisphosphate carboxylase large chain (Fragment)	0.496212121	0.527462121	1.062977099	0.041902243	PS
100	A0A061EH79	Ribulose bisphosphate carboxylase small chain	0.048913043	0.059782609	1.222222233	0.025721416	PS
101	A0A251SQD3	Putative sedoheptulose-1,7-bisphosphatase protein	0.036564626	0.027210884	0.744186043	0.007933091	PS
102	A0A118K612	Fructose-1,6-bisphosphatase, active site-containing protein	0.041556145	0.029177719	0.702127664	0.040545534	PS
103	M4QSX4	Transketolase	0.042838019	0.03480589	0.81249999	0.006533373	PS
104	A0A1R3K0M1	Uncharacterized protein	0.035346756	0.028635347	0.810126576	0.006073609	PS
105	A1BQW9	Transketolase (Fragment)	0.062222222	0.05	0.803571427	0.014172594	PS
106	A0A2C9VVI6	Uncharacterized protein	0.051762606	0.041499331	0.801724143	0.006184545	PS
107	A0A2G5E2S0	Uncharacterized protein	0.049531459	0.039268184	0.792792792	0.030337366	PS
108	A0A0L9UT98	Uncharacterized protein	0.051598174	0.040639269	0.787610609	0.022394205	PS
109	A0A1J7HK61	Uncharacterized protein	0.057674841	0.045413261	0.787401571	0.015217906	PS
110	A0A151UC86	Uncharacterized protein	0.06466877	0.049421661	0.764227638	0.017388554	PS
111	A0A0A0KBL8	Uncharacterized protein	0.040715884	0.029082774	0.714285713	0.00435682	PS
112	K4KJF0	ATP synthase subunit alpha, chloroplastic (Fragment)	0.119284294	0.093439364	0.783333331	0.021719951	PS
113	Q9MRM1	ATP synthase subunit beta (Fragment)	0.22515213	0.204868154	0.909909908	0.022525048	PS
114	A0A0A7DB11	ATP synthase subunit beta (Fragment)	0.216989843	0.194829178	0.897872339	0.011188829	PS
115	B7S4V7	ATP synthase subunit beta (Fragment)	0.283441793	0.249457701	0.88010204	0.016312603	PS
116	C0J9P4	ATP synthase subunit beta (Fragment)	0.352059925	0.308614232	0.876595745	0.02032551	PS
117	C0J9K5	ATP synthase subunit beta (Fragment)	0.336894587	0.294871795	0.875264271	0.019095051	PS
118	A0A0M3LBA7	ATP synthase subunit beta, chloroplastic	0.377333333	0.327333333	0.867491166	0.015556019	PS
119	Q9SCE9	ATP synthase subunit beta (Fragment)	0.305890227	0.265060241	0.866520789	0.011153748	PS
120	A0A1I7PIS3	ATP synthase subunit beta (Fragment)	0.356622998	0.30713246	0.86122449	0.008822824	PS
121	A0A200PYZ1	ATPase	0.098039215	0.084670232	0.863636369	0.042315662	PS
122	K3YUS1	Uncharacterized protein	0.027090695	0.021201413	0.782608688	0.007490434	PS
123	A0A1J6IVH5	Cytochrome b6-f complex iron-sulfur subunit	0.052631579	0.040935672	0.777777773	0.01613009	PS
124	M5W912	Ferredoxin--NADP reductase	0.066849817	0.041208791	0.616438352	0.024896164	PS
125	Q8HSZ7	Photosystem II CP43 reaction center protein (Fragment)	0.096296296	0.085596708	0.88888889	0.007966205	PS
126	A0A1U8LXQ6	Photosystem II CP47 reaction center protein	0.061139896	0.05388601	0.881355933	0.021663578	PS
127	B3SH56	Photosystem II CP43 reaction center protein (Fragment)	0.107655502	0.093301435	0.866666667	0.013235596	PS
128	A0A059TLB1	Photosystem II 32 kDa protein (Fragment)	0.358823529	0.307843137	0.857923498	0.035955539	PS
129	X2F7W5	Photosystem II	0.194523135	0.166194523	0.854368933	0.045911513	PS

130	Q8HRB5	Photosystem II	0.140698772	0.113314448	0.805369129	0.010142734	PS
131	A0A059P277	Photosystem II D2 protein	0.123701606	0.099150142	0.801526715	0.016608509	PS
132	A5BVF4	Uncharacterized protein	0.208333334	0.16025641	0.769230769	0.017295061	PS
133	D2XUU3	Chloroplast managanese stabilizing protein (Fragment)	0.226571768	0.168446026	0.743455497	0.004352853	PS
134	Q7M1Y7	Photosystem II oxygen-evolving complex protein 2 (Fragment)	0.306306306	0.207207207	0.676470588	0.029471316	PS
135	A4GKR3	Photosystem II subunit protein (Fragment)	0.425925926	0.277777778	0.652173913	0.011354999	PS
136	A0A251VJH6	Putative chlorophyll A-B binding protein	0.043256998	0.026717557	0.61764705	0.031375734	PS
137	W1PKM0	Uncharacterized protein	0.035670357	0.019680197	0.551724144	0.025481481	PS
138	A0A200QG47	Aminotransferase	0.060681629	0.047381546	0.78082192	0.001323897	PS
139	A0A2G5DDQ5	Uncharacterized protein	0.197093551	0.14986376	0.760368663	0.031375734	PS
140	M0TFM8	Uncharacterized protein	0.075386013	0.055404178	0.734939759	0.007933091	PS
141	A0A2I4DTC0	peroxisomal (S)-2-hydroxy-acid oxidase GLO1	0.070844687	0.047229791	0.666666665	0.001192882	PS
142	A9PJJ1	Uncharacterized protein	0.101036269	0.082901554	0.820512823	0.000265505	PS
143	Q19U04	NADH-dependent hydroxypyruvate reductase (Fragment)	0.136413641	0.111111111	0.814516131	0.001244101	PS
144	W9RXI1	Glycerate dehydrogenase	0.101254481	0.079749104	0.787610613	0.005820686	PS
145	W8TP69	Glycerate dehydrogenase-like protein	0.056131261	0.043177893	0.769230762	0.002570261	PS
146	A0A059D1J2	Uncharacterized protein	0.026722926	0.019690576	0.736842087	0.0241101	PS
147	A0A2I0WJ74	Glutathione reductase, chloroplastic	0.010600707	0.004711425	0.444444413	0.000562003	redox
148	A0A199UEL4	Catalase	0.023035231	0.040650407	1.764705881	1.30E-05	redox
149	A0A0U4JQY1	Catalase	0.027777778	0.040650407	1.463414646	0.001991489	redox
150	A0A1D8H339	2-Cys peroxiredoxin	0.093333333	0.072727272	0.779220775	0.013158072	redox
151	A0A1J6I7J0	2-cys peroxiredoxin bas1, chloroplastic	0.077403246	0.053682897	0.693548391	0.007090812	redox
152	A0A200R4I6	RNA recognition motif domain	0.060796646	0.037735849	0.620689654	0.005328129	RNA
153	A0A1W0W3M4	Uncharacterized protein	0.037414966	0.02154195	0.575757586	0.017797408	RNA
154	A0A1U8LIR6	2-methyl-6-phytyl-1,4-hydroquinone methyltransferase, chloroplastic-like isoform X2	0.016091954	0.025287356	1.571428607	0.023215158	secondary metabolism
155	Q8LSM9	Pathogenesis-related protein 5-1	0.016516517	0.04954955	2.999999976	0.000388171	stress
156	A0A200QCN5	Heat shock protein 70 family	0.031572556	0.060716454	1.923076927	0.000560116	stress
157	A0A0D9VBB6	Uncharacterized protein	0.026065163	0.042606516	1.6346154	0.004302991	stress
158	A0A2G5CU58	Uncharacterized protein	0.027486257	0.044477761	1.618181826	0.006046575	stress
159	B9I7J3	Heat shock protein 70 cognate	0.011616162	0.016161616	1.391304358	0.033471729	stress
160	Q42434	Luminal-binding protein	0.0249501	0.033932136	1.359999999	0.019890757	stress
161	A0A165ZM50	Uncharacterized protein	0.023481368	0.0316488	1.347826067	0.047420651	stress
162	A0A1D1XZB0	Heat shock cognate protein (Fragment)	0.046398046	0.061660561	1.328947364	0.001659064	stress

163	A0A0F7CS07	Heat shock cognate 70 kDa-like protein (Fragment)	0.033431662	0.042281219	1.264705875	0.008580919	stress
164	A0A0B2R5Y0	Heat shock cognate 70 kDa protein 2	0.049820237	0.061119671	1.226804115	0.001473119	stress
165	B9SP17	Heat shock protein, putative	0.054095827	0.064399794	1.190476183	0.003198202	stress
166	B2D2G5	70 kDa heat shock protein	0.046738572	0.055469954	1.186813196	0.005784564	stress
167	A0A1D1Y9S2	Heat shock protein 4 (Fragment)	0.048862115	0.057563588	1.178082202	0.017252806	stress
168	A0A067LJW8	Uncharacterized protein	0.061588331	0.072393301	1.17543859	0.007490434	stress
169	G7KWU8	Heat shock cognate 70 kDa protein	0.052469136	0.061213992	1.16666666	0.005784567	stress
170	A0A251UNC3	Putative heat shock protein	0.049897119	0.058127572	1.164948453	0.012886687	stress
171	J7EM74	HSP70	0.053846154	0.062564103	1.16190476	0.017540468	stress
172	A0A1S2YJT8	heat shock cognate 70 kDa protein 2	0.044684129	0.051874679	1.160919559	0.006122254	stress
173	W1NRJ1	Uncharacterized protein	0.051243024	0.059360731	1.158415838	0.007182329	stress
174	A0A2C9V2W2	Uncharacterized protein	0.054012346	0.062242798	1.152380946	0.011355004	stress
175	H1ZXA8	Heat shock protein 70 isoform 2	0.057971014	0.066252588	1.14285714	0.023215158	stress
176	M5W3N4	Uncharacterized protein	0.053543307	0.05984252	1.117647066	0.008049889	stress
177	A0A022PYG3	Uncharacterized protein	0.044989775	0.050102249	1.113636362	0.03935186	stress
178	I1IHD0	Uncharacterized protein	0.057613169	0.063271605	1.098214288	0.025386442	stress
179	A0A2G5EBY1	Uncharacterized protein	0.058551618	0.063687725	1.087719296	0.019441768	stress
180	A0A067DN94	Uncharacterized protein	0.06022409	0.049953314	0.829457359	0.028437309	stress
181	A0A1D1YEK4	Stromal heat shock-related protein, chloroplastic	0.071192834	0.058934465	0.827814565	0.011738599	stress
182	A0A218XKN3	Uncharacterized protein	0.055922501	0.042272127	0.755905521	0.001493531	stress
183	A0A175YQ55	Malate dehydrogenase	0.065774805	0.049052397	0.745762704	0.00257026	TCA / org
184	A0A2I0HJ74	Uncharacterized protein	0.032967033	0.059523809	1.805555549	0.008855845	transport

[a] Protein ID, according to UniProtKB/Swiss-Prot database

[d] Function, protein function categorized using MapMan bin codes. ETC, mitochondrial electron transport chains; C1, one-carbon; TCA, tricarboxylic acid; CHO, carbohydrate; OPP, oxidative pentose phosphate.

Table S7. List of Proteins Identified in Leaf of *Clematis terniflora* DC. in VC

No.	Protein ID[a]	Description	VC at starting point abundance	VC after HUV-B+D abundance	Fold changs	P-value	Annotation
1	A0A022QVC8	Adenosylhomocysteinase	0.020618557	0.032989691	1.633333339	0.008970902	amino acid metabolism
2	A0A1U8AZ67	probable LL-diaminopimelate aminotransferase, chloroplastic isoform X2	0.01511335	0.033676976	0.77777777	0.016130074	amino acid metabolism
3	A0A067L6G5	Uncharacterized protein	0.024400871	0.011754828	1.142857139	0.047420675	amino acid metabolism
4	B9RET4	Cysteine synthase	0.054358974	0.02788671	1.075471696	0.01613009	amino acid metabolism
5	A0A1Z5S503	Serine hydroxymethyltransferase	0.066276803	0.054592164	0.754901963	0.00334135	C1-metabolism
6	A0A103XZB1	Serine hydroxymethyltransferase (Fragment)	0.070648683	0.058461538	0.772727272	0.04126871	C2-metabolism
7	A0A2G5DC38	Uncharacterized protein	0.108753316	0.189247312	1.333333328	0.000510727	cell
8	Q9ZQT0	Actin (Fragment)	0.12688172	0.050032489	1.491525423	0.000942743	cell
9	A0A200Q637	Fructose-bisphosphate aldolase	0.031657356	0.145004421	1.264705873	0.015799848	glycolysis
10	G3EXN4	Enolase	0.035976505	0.040037244	1.163265292	0.01613009	glycolysis
11	A0A167NYG6	Phosphoenolpyruvate carboxylase	0.013085399	0.022845275	1.26315791	0.019441761	glycolysis
12	A0A2G5CS13	Uncharacterized protein	0.017653167	0.04185022	1.29411767	0.030694537	glycolysis
13	A0A200RAD1	UTP--glucose-1-phosphate uridylyltransferase	0.040169133	0.029850746	0.701754396	0.022269891	glycolysis
14	A0A067KU12	UTP--glucose-1-phosphate uridylyltransferase	0.041222459	0.016528926	0.724137935	0.045300145	glycolysis
15	V4SJ20	Uncharacterized protein	0.042914172	0.028188866	0.883720931	0.024110111	major CHO metabolism
16	A0A0D6QRT0	ATP synthase subunit beta	0.06538237	0.058823529	0.812500006	0.001756611	mitochondrial electron transport / ATP synthesis
17	H8XXL4	ATP synthase subunit alpha (Fragment)	0.041920216	0.037924152	1.403225805	0.016777902	mitochondrial electron transport / ATP synthesis
18	A0A1D1YMH1	Ferredoxin-dependent glutamate synthase, chloroplastic	0.02296023	0.014537265	0.821428587	0.003811655	N-metabolism.ammonia metabolism
19	A0A0G2UEW3	Ferredoxin-dependent glutamate synthase	0.019246519	0.021216098	0.755319152	0.005062734	N-metabolism.ammonia metabolism
20	A0A2G5EWR5	Uncharacterized protein	0.027340332	0.018222768	0.775999992	0.007762603	N-metabolism.ammonia metabolism
21	B9H8U4	Ferredoxin-dependent glutamate synthase family protein	0.023341523	0.019619865	0.780701745	0.014732454	N-metabolism.ammonia metabolism
22	A0A200PTK6	Class II glutamine amidotransferase domain	0.024524831	0.053123176	0.799999997	0.018411105	N-metabolism.ammonia metabolism
23	A0A022RHN8	Uncharacterized protein	0.044854881	0.018860189	0.764705884	0.025721419	not assigned
24	A0A0D3B1C7	Uncharacterized protein	0.015561016	0.01308699	1.578947364	0.005328128	OPP
25	W1P8B5	Uncharacterized protein	0.008468052	0.034300792	1.545454609	0.0132356	OPP

26	A0A2G5EPB1	6-phosphogluconate dehydrogenase, decarboxylating	0.032854209	0.030612245	1.479166667	0.001898447	OPP
27	A0A200QQ04	6-phosphogluconate dehydrogenase, decarboxylating	0.021088435	0.024570025	1.451612911	0.040545534	OPP
28	A0A022R419	Uncharacterized protein	0.022491349	0.048596851	0.820512841	0.002192132	protein
29	W1NKM9	Uncharacterized protein	0.033193081	0.018454441	1.126760563	0.033471727	protein
30	A0A1J3H2G2	Ubiquitin-NEDD8-like protein RUB1 (Fragment)	0.297385621	0.037400655	1.263736263	0.019978779	protein
31	D7M7T7	Predicted protein	0.051046452	0.046380581	1.469999995	1.94E-05	protein
32	W9QWD3	RuBisCO large subunit-binding protein subunit beta	0.059384482	0.009803922	0.7810219	0.000178521	protein
33	A0A251RQT3	Putative peptidyl-prolyl cis-trans isomerase protein	0.018853696	0.057471264	0.519999996	0.001057564	protein
34	A0A1U7Z848	ruBisCO large subunit-binding protein subunit beta, chloroplastic	0.072249589	0.375816993	0.795454547	0.005288625	protein
35	A0A2H3ZZH5	LOW QUALITY PROTEIN: elongation factor 2-like	0.011466983	0.060755337	1.827586185	0.001057564	protein
36	W9S3Y1	Nascent polypeptide-associated complex subunit alpha-like protein	0.036124795	0.075038285	1.681818186	0.010636628	protein
37	A0A2I0JKM9	Uncharacterized protein (Fragment)	0.067901235	0.0209569	0.681818181	0.024896164	protein
38	A0A175YFY1	Ribosomal protein	0.021012417	0.046296296	0.727272732	0.013235592	protein
39	Q01517	Fructose-bisphosphate aldolase 2, chloroplastic	0.050620821	0.051413882	0.754716979	0.004356821	PS
40	A0A2I0APB2	Fructose-bisphosphate aldolase, chloroplastic	0.066838046	0.048945148	0.769230776	0.009678952	PS
41	M1AVJ2	Fructose-bisphosphate aldolase	0.063291139	0.015281758	0.773333332	0.032505367	PS
42	A0A0A0L177	Uncharacterized protein	0.047734628	0.038204393	1.254237296	0.016010965	PS
43	A0A218X6H3	Glyceraldehyde-3-phosphate dehydrogenase	0.097359736	0.05987055	1.194915258	0.039161391	PS
44	U5DGY2	Phosphoglycerate kinase	0.122661123	0.106776181	0.915254233	0.017947908	PS
45	S8E173	Phosphoglycerate kinase	0.115674196	0.133744856	0.923076927	0.022645999	PS
46	A0A200PMV3	Phosphoglycerate kinase	0.143347051	0.114822547	0.933014355	0.032532525	PS
47	A0A2G5D2J7	Phosphoglycerate kinase	0.12526096	0.116336634	0.916666667	0.037590109	PS
48	A0A103Y6F3	ATPase, AAA-type, core	0.041666667	0.015607581	0.380952381	0.002890007	PS
49	B8LQV7	Uncharacterized protein	0.010033445	0.032478632	1.555555512	0.01944178	PS
50	W9SAI5	RuBisCO large subunit-binding protein subunit alpha	0.027350427	0.027404086	1.187499996	0.021311634	PS
51	A0A251VPF5	Putative chaperonin-60alpha	0.024912805	0.022530329	1.100000019	0.02411008	PS
52	M0U7I3	Uncharacterized protein	0.019064125	0.112266112	1.181818171	0.025721436	PS
53	V5K6K1	Ribulose bisphosphate carboxylase large	0.489379085	0.528344671	0.921535894	0.005324865	PS

		chain (Fragment)					
54	A0A248SN48	Ribulose bisphosphate carboxylase large chain (Fragment)	0.591080877	0.346181299	0.893861892	0.007559783	PS
55	Q9GD27	Ribulose bisphosphate carboxylase large chain (Fragment)	0.368308351	0.529137529	0.93992248	0.008035151	PS
56	A0A291L206	Ribulose bisphosphate carboxylase large chain (Fragment)	0.568764569	0.459925093	0.930327869	0.008797493	PS
57	Q8WJX3	Ribulose bisphosphate carboxylase large chain (Fragment)	0.492883895	0.523914643	0.9331307	0.009561053	PS
58	O47280	Ribulose bisphosphate carboxylase large chain (Fragment)	0.576894776	0.507865169	0.908163265	0.009819467	PS
59	E5G0I9	Ribulose bisphosphate carboxylase large chain (Fragment)	0.557303371	0.420350877	0.911290323	0.009874239	PS
60	O03613	Ribulose bisphosphate carboxylase large chain	0.464561403	0.478841871	0.904833838	0.010632845	PS
61	Q8HVI8	Ribulose bisphosphate carboxylase large chain (Fragment)	0.516703786	0.530555556	0.926724137	0.011342897	PS
62	D6MYK4	Ribulose bisphosphate carboxylase large chain	0.581944444	0.399843505	0.911694511	0.012633992	PS
63	A0A0N7FMB3	Ribulose bisphosphate carboxylase large chain (Fragment)	0.449921753	0.61627907	0.888695652	0.012886687	PS
64	A0A120KLC1	Ribulose bisphosphate carboxylase large chain (Fragment)	0.670542636	0.461052631	0.919075144	0.01613009	PS
65	Q9TN58	Ribulose bisphosphate carboxylase large chain	0.505263158	0.494444444	0.9125	0.0168857	PS
66	D6MYJ0	Ribulose bisphosphate carboxylase large chain	0.548611111	0.402905199	0.901265823	0.018709775	PS
67	B1NFY5	Ribulose bisphosphate carboxylase large chain (Fragment)	0.436544342	0.605769231	0.922942208	0.019086888	PS
68	A0A249Y5N8	Ribulose bisphosphate carboxylase large chain (Fragment)	0.662660256	0.564160972	0.914147521	0.019094742	PS
69	I2BGM7	Ribulose bisphosphate carboxylase large chain (Fragment)	0.600607441	0.499627144	0.93931732	0.019386718	PS
70	G8EEM9	Ribulose bisphosphate carboxylase large chain (Fragment)	0.530201342	0.593258427	0.94233474	0.020345144	PS
71	F2X4H9	Ribulose bisphosphate carboxylase large chain (Fragment)	0.651685393	0.400630915	0.910344828	0.02428999	PS
72	A0A076GVP1	Ribulose-1,5-bisphosphate carboxylase/oxygenase large unit (Fragment)	0.431125132	0.623582766	0.929268291	0.025673161	PS
73	I2E9A4	Ribulose bisphosphate carboxylase large	0.685563114	0.575233981	0.909592061	0.026716	PS

		chain (Fragment)					
74	E7DMY2	Ribulose bisphosphate carboxylase large chain (Fragment)	0.624910007	0.538888889	0.920506912	0.027085457	PS
75	D6MYJ6	Ribulose bisphosphate carboxylase large chain	0.592361111	0.621902478	0.909730363	0.027453263	PS
76	L0N8B1	Ribulose bisphosphate carboxylase large chain (Fragment)	0.677058353	0.54245614	0.918536009	0.02770844	PS
77	Q5I221	Ribulose bisphosphate carboxylase large chain	0.59368421	0.356895357	0.913711584	0.028022264	PS
78	A0A142DPV4	Ribulose bisphosphate carboxylase large chain	0.378378378	0.597701149	0.943223444	0.028241338	PS
79	A0A0U4D534	Ribulose bisphosphate carboxylase large chain (Fragment)	0.655172414	0.566951567	0.912280702	0.029349579	PS
80	Q33032	Ribulose bisphosphate carboxylase large chain (Fragment)	0.613960114	0.531595577	0.923433875	0.029356033	PS
81	Q6VW13	Ribulose bisphosphate carboxylase large chain (Fragment)	0.583728278	0.599002849	0.910690122	0.029702177	PS
82	C0J9R6	Ribulose bisphosphate carboxylase large chain (Fragment)	0.6502849	0.483801296	0.921139102	0.029930206	PS
83	Q8ME51	Ribulose bisphosphate carboxylase large chain (Fragment)	0.534197264	0.415895062	0.905660377	0.03066008	PS
84	A0A142I7F3	Ribulose bisphosphate carboxylase large chain (Fragment)	0.455246913	0.635103926	0.913559322	0.032900038	PS
85	Q8MFT9	Ribulose bisphosphate carboxylase large chain (Fragment)	0.688221709	0.466183575	0.922818792	0.033555994	PS
86	A0A291L2S1	Ribulose bisphosphate carboxylase large chain (Fragment)	0.51610306	0.573611111	0.903276131	0.037860558	PS
87	D6MYI7	Ribulose bisphosphate carboxylase large chain	0.622222222	0.548245614	0.921875001	0.038379457	PS
88	Q7GH77	Ribulose bisphosphate carboxylase large chain (Fragment)	0.59502924	0.572801182	0.921375922	0.040577401	PS
89	Q7YJP8	Ribulose bisphosphate carboxylase large chain (Fragment)	0.624538064	0.476718404	0.917159763	0.044598321	PS
90	Q7YJM6	Ribulose bisphosphate carboxylase large chain (Fragment)	0.513673319	0.615730337	0.928057554	0.044924811	PS
91	R4QE58	Ribulose bisphosphate carboxylase large chain (Fragment)	0.665168539	0.30942029	0.925675676	0.046110728	PS
92	Q49M31	Ribulose bisphosphate carboxylase large chain (Fragment)	0.329710145	0.444444444	0.938461538	0.047420655	PS
93	D6MYK9	Ribulose bisphosphate carboxylase large chain	0.475694444	0.015873016	0.934306569	0.048463578	PS

94	A0A200PYZ1	ATPase	0.083778966	0.450980392	1.308510634	0.000785043	PS
95	A0A0A7LIM5	Cytochrome f	0.064182194	0.038011696	1.306451624	0.000685233	PS
96	A0A1J6IVH5	Cytochrome b6-f complex iron-sulfur subunit	0.049707602	0.109625668	0.764705877	0.047420656	PS
97	A0A1R3GDQ7	Photosystem I PsaN, reaction centre subunit N	0.136452242	0.097184378	1.228571428	0.000347891	PS
98	A0A1V0J295	Photosystem I P700 chlorophyll a apoprotein A2	0.088101725	0.171875	1.103092786	0.011056498	PS
99	I0YVS7	PSI-D1	0.151041667	0.083850932	1.137931034	0.036277828	PS
100	G8HAA9	PLP-dependent aminotransferase	0.027083333	0.167641325	0.769230769	0.021311647	PS
101	A0A2H3Z4Q2	Glycine cleavage system P protein	0.016346154	0.019732206	0.686274534	0.001323898	PS
102	J3L3E8	Glycine cleavage system P protein	0.024312896	0.020872865	0.811594201	0.007966203	PS
103	A0A200QMI6	Glycine cleavage system P protein	0.024667932	0.020833333	0.84615385	0.036277838	PS
104	A0A2G5DDQ5	Uncharacterized protein	0.211625795	0.058128974	0.763948497	0.002013462	PS
105	A0A2I4DTC0	peroxisomal (S)-2-hydroxy-acid oxidase GLO1	0.071752952	0.066303361	0.810126581	0.010636627	PS
106	M0TFM8	Uncharacterized protein	0.076294278	0.011217949	0.869047619	0.014172596	PS
107	A0A059D1J2	Uncharacterized protein	0.028129395	0.161671208	0.749999982	0.00749043	PS
108	A0A151U9E4	Uncharacterized protein	0.031565656	0.021097046	0.600000006	0.007490436	redox
109	A0A2I0WJ74	Glutathione reductase, chloroplastic	0.008244994	0.018939394	0.714285703	0.047420656	redox
110	A0A0N6ZBJ9	Catalase	0.032520325	0.059620596	1.583333319	0.000151011	redox
111	A0A022QUS5	Catalase	0.041327913	0.0399729	1.442622945	0.000672594	redox
112	A0A0U4JQY1	Catalase	0.025067751	0.005889281	1.594594592	0.012500998	redox
113	W9QII5	Peroxiredoxin Q	0.037558685	0.051490515	0.70833333	0.024896162	redox
114	A0A2G5DW13	Uncharacterized protein	0.061594203	0.026604069	0.676470587	0.017704295	redox
115	A0A140CWP1	4-hydroxy-3-methylbut-2-en-1-yl diphosphate synthase	0.006756757	0.017379679	2.533333398	0.001898447	secondary metabolism
116	A0A1V0QSG3	HDS (Fragment)	0.007575758	0.041666667	2.294117647	0.003650262	secondary metabolism
117	A0A1U8LIR6	2-methyl-6-phytyl-1,4-hydroquinone methyltransferase, chloroplastic-like isoform X2	0.022988506	0.017117117	1.250000011	0.024110111	secondary metabolism
118	A0A2G5CER3	Uncharacterized protein (Fragment)	0.011164274	0.028735632	1.285714323	0.016130073	signalling
119	Q8LSM9	Pathogenesis-related protein 5-1	0.015015015	0.014354067	3.49999996	0.000364348	stress
120	A0A2G5EQF7	Uncharacterized protein	0.042380952	0.078674949	1.856375195	2.77E-05	stress
121	H1ZXA8	Heat shock protein 70 isoform 2	0.057453416	0.042042042	1.36936938	3.34E-05	stress
122	A0A2I4FU77	luminal-binding protein	0.023023023	0.085949178	1.826086945	4.52E-05	stress
123	A0A165A740	Uncharacterized protein	0.056801196	0.082117774	1.5131579	6.34E-05	stress

124	A0A067LJW8	Uncharacterized protein	0.062128579	0.082417582	1.321739124	7.81E-05	stress
125	A0A1D1XZB0	Heat shock cognate protein (Fragment)	0.049450549	0.064417178	1.666666681	9.89E-05	stress
126	A0A022PYG3	Uncharacterized protein	0.044989775	0.072016461	1.431818185	0.000136555	stress
127	A0A251UNC3	Putative heat shock protein	0.047839506	0.073732719	1.505376345	0.000199893	stress
128	A0A1J3FXE7	Putative mediator of RNA polymerase II transcription subunit 37e	0.050691244	0.044845123	1.454545455	0.000202627	stress
129	A0A176WEL9	Uncharacterized protein	0.033287101	0.064814815	1.347222223	0.000235616	stress
130	A0A2H3ZD96	heat shock cognate 70 kDa protein 2-like	0.04372428	0.073673364	1.48235294	0.000341065	stress
131	A0A140CZC8	Heat shock cognate 70 kDa protein 2-like protein	0.052035034	0.076115485	1.415841581	0.000358735	stress
132	M5W3N4	Uncharacterized protein	0.05511811	0.070066976	1.380952383	0.000375519	stress
133	A0A1S3E0E2	heat shock cognate 70 kDa protein 2-like isoform X3	0.048943844	0.080246914	1.431578937	0.000394036	stress
134	I1IHD0	Uncharacterized protein	0.062757202	0.076646091	1.278688526	0.000424225	stress
135	G7KWU8	Heat shock cognate 70 kDa protein	0.052983539	0.063174114	1.446601937	0.000503846	stress
136	A0A1S2YJT8	heat shock cognate 70 kDa protein 2	0.042116076	0.061897514	1.500000013	0.000510727	stress
137	D8RBE2	Uncharacterized protein	0.03957382	0.072419106	1.564102589	0.000541944	stress
138	M5X5S2	Uncharacterized protein	0.049820237	0.080636878	1.453608229	0.000541944	stress
139	A0A2G5EBY1	Uncharacterized protein	0.059578839	0.032206119	1.353448282	0.000574363	stress
140	S8EAM3	Heat shock protein hsp70 (Fragment)	0.019323671	0.064201335	1.666666672	0.000608185	stress
141	B2D2G5	70 kDa heat shock protein	0.044684129	0.068648266	1.436781622	0.000685233	stress
142	A0A103YD73	Heat shock protein 70, conserved site-containing protein	0.044585988	0.048475762	1.539682523	0.00070509	stress
143	A0A2G5CU58	Uncharacterized protein	0.024487756	0.077435897	1.979591814	0.000709818	stress
144	J7EM74	HSP70	0.055897436	0.064989518	1.385321104	0.000716276	stress
145	A0A0D3AEW4	Uncharacterized protein	0.049266247	0.071905495	1.31914895	0.00082769	stress
146	A0A0B2R5Y0	Heat shock cognate 70 kDa protein 2	0.049820237	0.073559671	1.443298952	0.00085615	stress
147	A0A2C9V2W2	Uncharacterized protein	0.056584362	0.049655851	1.3	0.000912048	stress
148	A0A0F7CS07	Heat shock cognate 70 kDa-like protein (Fragment)	0.033431662	0.048975512	1.485294126	0.000912048	stress
149	A0A1S3YP64	luminal-binding protein 4-like	0.026486757	0.071029934	1.849056592	0.001121323	stress
150	W1NRJ1	Uncharacterized protein	0.048198884	0.074074074	1.47368422	0.001121324	stress
151	A0A1D5XSS6	Uncharacterized protein	0.051440329	0.046115288	1.439999995	0.001142619	stress
152	A0A0D9VBB6	Uncharacterized protein	0.025062657	0.067386831	1.840000003	0.001184643	stress
153	A9S0A3	Predicted protein	0.047325103	0.067395264	1.42391304	0.001243906	stress
154	A0A200QCN5	Heat shock protein 70 family	0.03339405	0.050071531	2.0181818	0.00148204	stress
155	A0A0D2Q3K9	Uncharacterized protein	0.035288507	0.054368932	1.418918918	0.001493531	stress

156	A0A2G5DZJ7	Uncharacterized protein	0.027184466	0.074642127	2	0.00155487	stress
157	A0A0K9Q3W1	70 kDa heat shock protein	0.053169734	0.064814815	1.403846156	0.001756611	stress
158	A9RCV9	Predicted protein	0.044753086	0.070473251	1.448275869	0.002056281	stress
159	B9NBF4	Heat shock protein 70 cognate	0.052469136	0.073959938	1.343137248	0.002708655	stress
160	A0A251SC78	Putative heat shock cognate 70 kDa protein 2	0.051361068	0.05306543	1.439999999	0.002910811	stress
161	A0A2G5DR54	Uncharacterized protein	0.041731066	0.057859703	1.271604946	0.00532813	stress
162	C9WCK6	Heat shock protein 70	0.043522785	0.019191919	1.329411769	0.007762601	stress
163	B9I7J3	Heat shock protein 70 cognate	0.011616162	0.00808781	1.652173888	0.010636631	stress
164	A0A2G5F615	Uncharacterized protein	0.011554015	0.052552553	0.699999965	0.013235587	stress
165	A0A068TS20	Dihydrolipoyl dehydrogenase	0.014521452	0.062857143	1.363636364	0.01613009	TCA / org
166	A0A2G5CYH0	Succinate--CoA ligase [ADP-forming] subunit beta, mitochondrial	0.012789768	0.01980198	1.68749997	0.005328128	TCA / org
167	A0A2G9HCF9	Mitochondrial ADP/ATP carrier protein	0.020779221	0.021582734	2.249999972	6.52E-05	transport
168	A0A2I0HJ74	Uncharacterized protein	0.031135531	0.046753247	1.941176475	0.000268047	transport

[a] Protein ID, according to UniProtKB/Swiss-Prot database

[d] Function, protein function categorized using MapMan bin codes. ETC, mitochondrial electron transport chains; C1, one-carbon; TCA, tricarboxylic acid; CHO, carbohydrate; OPP, oxidative pentose phosphate.

Table S8. List of Proteins Identified in Leaf of *Clematis terniflora* DC. at Starting Point

No.	Protein ID[a]	Description	VV	VC	Fold changs	P-value	Annotation
1	A0A1U8AZ67	probable LL-diaminopimelate aminotransferase, chloroplastic isoform X2	0.018471873	0.01511335	0.818181798	0.01613009	amino acid metabolism.synthesis
2	Q9ZQT0	Actin (Fragment)	0.169892473	0.12688172	0.746835443	0.035407551	cell
3	A0A2G3DEA2	3-oxo-Delta(4,5)-steroid 5-beta-reductase	0.026190476	0.038095238	1.454545461	0.02411011	development
4	A0A067KU12	UTP--glucose-1-phosphate uridylyltransferase	0.026297086	0.041222459	1.567567583	0.017547374	glycolysis
5	A0A200RAD1	UTP--glucose-1-phosphate uridylyltransferase	0.028188866	0.040169133	1.42499998	0.043439505	glycolysis
6	G3EXN4	Enolase	0.030837004	0.035976505	1.166666675	0.024896167	glycolysis
7	A0A142NIP8	Glyceraldehyde-3-phosphate dehydrogenase	0.080118694	0.088031652	1.098765439	0.01613009	glycolysis
8	A0A200QPK9	Phosphoglycerate kinase	0.167913549	0.183707398	1.094059408	0.036852392	glycolysis
9	A0A2I0XF37	Phosphoglycerate kinase, cytosolic	0.133000831	0.145469659	1.09375	0.016010967	glycolysis
10	A0A0D6QRT0	ATP synthase subunit beta	0.049620549	0.06538237	1.317647049	0.011875112	mitochondrial electron transport / ATP synthesis
11	H8XXL4	ATP synthase subunit alpha (Fragment)	0.062880324	0.041920216	0.666666668	0.008035153	mitochondrial electron transport / ATP synthesis

12	A0A0B0MQT9	Glutamine synthetase	0.05787037	0.040123457	0.693333332	0.033231257	N-metabolism
13	A0A022RHN8	Uncharacterized protein	0.032541777	0.044854881	1.378378379	0.024896167	not assigned
14	A0A2G3BP94	Uncharacterized protein	0.023504274	0.012820513	0.545454549	0.007490436	not assigned
15	W9QWD3	RuBisCO large subunit-binding protein subunit beta	0.045080191	0.059384482	1.317307686	0.003593113	protein
16	A0A1U7Z848	ruBisCO large subunit-binding protein subunit beta, chloroplastic	0.055281883	0.072249589	1.306930686	0.005097542	protein
17	A0A1J3H2G2	Ubiquitin-NEDD8-like protein RUB1 (Fragment)	0.22875817	0.297385621	1.3	0.004952037	protein
18	A0A2G5F864	Uncharacterized protein	0.066823899	0.061320755	0.917647068	0.024896171	protein
19	W9SL34	Elongation factor Ts, mitochondrial	0.006289308	0.005031446	0.799999947	0.047420656	protein
20	W1NKM9	Uncharacterized protein	0.04628331	0.033193081	0.717171719	0.000151011	protein
21	W9S3Y1	Nascent polypeptide-associated complex subunit alpha-like protein	0.055829228	0.036124795	0.647058821	0.032677921	protein
22	Q8M9K2	Ribulose-bisphosphate carboxylase (Fragment)	0.794117647	1.460784313	1.839506173	0.001050578	PS
23	A0A068TPY5	Uncharacterized protein	0.029462738	0.045060658	1.529411755	0.007875643	PS
24	A1X444	Ribulose-1,5-bisphosphate carboxylase/oxygenase large subunit (Fragment)	0.268156425	0.38547486	1.4375	0.000626981	PS
25	A0A0A0QCC3	Ribulose bisphosphate carboxylase large chain (Fragment)	0.264444445	0.375555556	1.420168067	6.52E-05	PS
26	A0A067KC46	Ribulose bisphosphate carboxylase small chain	0.063180828	0.089324619	1.4137931	0.022394207	PS
27	A0A061EH79	Ribulose bisphosphate carboxylase small chain	0.048913043	0.06884058	1.407407421	0.000388171	PS
28	F6JSX0	Ribulose bisphosphate carboxylase large chain (Fragment)	0.211509716	0.291479821	1.378091872	0.000383236	PS
29	Q36373	Ribulose bisphosphate carboxylase large chain (Fragment)	0.256124722	0.349665924	1.365217392	4.13E-05	PS
30	Q31895	Ribulose bisphosphate carboxylase large chain (Fragment)	0.285612535	0.388888889	1.361596011	0.000131532	PS
31	Q95631	Ribulose bisphosphate carboxylase large chain (Fragment)	0.331201137	0.44633973	1.347639486	0.000164838	PS
32	Q37220	Ribulose bisphosphate carboxylase large chain (Fragment)	0.363161819	0.486204325	1.338809036	0.000256594	PS
33	J9QER6	Ribulose bisphosphate carboxylase large chain (Fragment)	0.276934201	0.368763557	1.33159269	0.000771053	PS
34	Q37279	Ribulose bisphosphate carboxylase large chain (Fragment)	0.303221288	0.403361345	1.330254044	0.000597135	PS

35	Q32553	Ribulose bisphosphate carboxylase large chain (Fragment)	0.332628611	0.440451022	1.324152544	0.000489559	PS
36	A0A291L2S1	Ribulose bisphosphate carboxylase large chain (Fragment)	0.390499195	0.51610306	1.321649485	0.000868581	PS
37	Q7YMT1	Ribulose bisphosphate carboxylase large chain	0.30967293	0.407098121	1.314606741	0.000392927	PS
38	C1JAT3	Ribulose-1,5-bisphosphate carboxylase/oxygenase large subunit (Fragment)	0.350961539	0.459935897	1.310502282	0.000245957	PS
39	Q4VI78	Ribulose bisphosphate carboxylase large chain (Fragment)	0.357562408	0.468428781	1.310061603	0.000339816	PS
40	C7A5Z4	Ribulose bisphosphate carboxylase large chain	0.310175439	0.404210526	1.303167421	0.001111786	PS
41	B1NL21	Ribulose bisphosphate carboxylase large chain (Fragment)	0.310606061	0.403198653	1.298102979	0.000120298	PS
42	Q3T5L7	Ribulose bisphosphate carboxylase large chain (Fragment)	0.399283154	0.517562724	1.296229803	0.00351148	PS
43	A4H1D1	Ribulose bisphosphate carboxylase large chain (Fragment)	0.285493827	0.368827161	1.291891891	0.000269795	PS
44	Q9XQR0	Ribulose bisphosphate carboxylase large chain	0.266666666	0.343157895	1.286842106	0.000253915	PS
45	V5T6A6	Ribulose bisphosphate carboxylase large chain (Fragment)	0.465465465	0.596096096	1.280645162	0.009372692	PS
46	A0A142DPV4	Ribulose bisphosphate carboxylase large chain	0.295911296	0.378378378	1.278688526	8.82E-05	PS
47	O03628	Ribulose bisphosphate carboxylase large chain	0.261754386	0.333333333	1.273458445	0.001164875	PS
48	Q8WL19	Ribulose bisphosphate carboxylase large chain (Fragment)	0.277065527	0.351851852	1.269922877	4.96E-05	PS
49	A0A0F7EV98	Ribulose bisphosphate carboxylase large chain (Fragment)	0.307692308	0.390313391	1.26851852	0.000156892	PS
50	A0A0N7FMB3	Ribulose bisphosphate carboxylase large chain (Fragment)	0.356025039	0.449921753	1.263736265	0.00074818	PS
51	B8PRL7	Ribulose bisphosphate carboxylase large chain (Fragment)	0.306267806	0.386039886	1.260465117	0.001186001	PS
52	D6MYK9	Ribulose bisphosphate carboxylase large chain	0.377777778	0.475694444	1.259191176	2.52E-05	PS
53	C7AQV0	Ribulose bisphosphate carboxylase large chain (Fragment)	0.247050147	0.310471976	1.256716419	0.000204189	PS
54	Q9TN58	Ribulose bisphosphate carboxylase large chain	0.402105263	0.505263158	1.256544502	0.000394173	PS

55	D6MYJ0	Ribulose bisphosphate carboxylase large chain	0.4375	0.548611111	1.253968253	0.001478799	PS
56	A0A1Q1G8P4	Ribulose bisphosphate carboxylase large chain (Fragment)	0.120676968	0.15084621	1.25	0.03285186	PS
57	F2X4H9	Ribulose bisphosphate carboxylase large chain (Fragment)	0.521348315	0.651685393	1.249999998	0.000956479	PS
58	Q8ME51	Ribulose bisphosphate carboxylase large chain (Fragment)	0.427645788	0.534197264	1.24915825	0.001373968	PS
59	Q32701	Ribulose bisphosphate carboxylase large chain	0.456140351	0.569122807	1.247692309	0.000921331	PS
60	A0A249Y5N8	Ribulose bisphosphate carboxylase large chain (Fragment)	0.53125	0.662660256	1.247360482	0.00079542	PS
61	D6MYJ6	Ribulose bisphosphate carboxylase large chain	0.475	0.592361111	1.247076024	0.001217217	PS
62	D6MYK4	Ribulose bisphosphate carboxylase large chain	0.467361111	0.581944444	1.245170877	0.000635014	PS
63	Q3I534	Ribulose bisphosphate carboxylase large chain (Fragment)	0.380743982	0.473377097	1.24329502	0.000438979	PS
64	Q5I221	Ribulose bisphosphate carboxylase large chain	0.477894737	0.59368421	1.242290749	0.001239959	PS
65	Q8WJX3	Ribulose bisphosphate carboxylase large chain (Fragment)	0.397003745	0.492883895	1.241509433	0.000170815	PS
66	Q7GH77	Ribulose bisphosphate carboxylase large chain (Fragment)	0.479532163	0.59502924	1.24085366	0.001203435	PS
67	D6MYI7	Ribulose bisphosphate carboxylase large chain	0.502083333	0.622222222	1.239280774	0.001054256	PS
68	A0A1C9HG87	Ribulose bisphosphate carboxylase large chain (Fragment)	0.526570048	0.65136876	1.237003059	0.001171216	PS
69	Q8MFT9	Ribulose bisphosphate carboxylase large chain (Fragment)	0.556581986	0.688221709	1.236514523	0.000355515	PS
70	V5K6K1	Ribulose bisphosphate carboxylase large chain (Fragment)	0.39624183	0.489379085	1.235051546	0.001594785	PS
71	R4QE58	Ribulose bisphosphate carboxylase large chain (Fragment)	0.538576779	0.665168539	1.235048677	0.001437321	PS
72	Q33032	Ribulose bisphosphate carboxylase large chain (Fragment)	0.497150997	0.613960114	1.234957021	0.000843591	PS
73	I2E9A4	Ribulose bisphosphate carboxylase large chain (Fragment)	0.555555556	0.685563114	1.234013605	0.002092743	PS
74	S6CMZ7	Ribulose bisphosphate carboxylase large chain (Fragment)	0.489187174	0.603281133	1.233231707	0.000953502	PS
75	Q8MCW3	Ribulose bisphosphate carboxylase large chain (Fragment)	0.42303433	0.521594685	1.232984293	0.003689627	PS

76	O47280	Ribulose bisphosphate carboxylase large chain (Fragment)	0.46799117	0.576894776	1.232704404	0.000222032	PS
77	E7DMY2	Ribulose bisphosphate carboxylase large chain (Fragment)	0.507559395	0.624910007	1.231205673	0.000966205	PS
78	A0A2H4UGV5	Ribulose-1,5-bisphosphate carboxylase/oxygenase large subunit (Fragment)	0.454545455	0.559497413	1.230894308	0.002227084	PS
79	L0N8B1	Ribulose bisphosphate carboxylase large chain (Fragment)	0.550759393	0.677058353	1.229317852	0.001222049	PS
80	Q31889	Ribulose bisphosphate carboxylase large chain (Fragment)	0.322829131	0.396358543	1.227765727	0.000447238	PS
81	Q37309	Ribulose bisphosphate carboxylase large chain (Fragment)	0.331641286	0.406937394	1.227040816	0.013848782	PS
82	A0A2H5NKV2	Uncharacterized protein	0.183413078	0.224880383	1.226086956	0.01660851	PS
83	C7AQV2	Ribulose bisphosphate carboxylase large chain (Fragment)	0.324483776	0.397492625	1.224999999	0.001256611	PS
84	C7SN35	Ribulose bisphosphate carboxylase large chain (Fragment)	0.251141553	0.307458143	1.224242423	0.021853935	PS
85	A0A0F7G5Y0	Ribulose bisphosphate carboxylase large chain (Fragment)	0.466027178	0.568345324	1.219554032	0.000443454	PS
86	A0A024HRQ0	Ribulose bisphosphate carboxylase large chain (Fragment)	0.408551069	0.496437054	1.215116278	0.000699625	PS
87	P92447	Ribulose bisphosphate carboxylase large chain (Fragment)	0.373845061	0.454157783	1.214828896	0.000868245	PS
88	J9QG76	Ribulose bisphosphate carboxylase large chain (Fragment)	0.449023861	0.543745481	1.21095008	0.000883555	PS
89	Q8HUZ9	Ribulose bisphosphate carboxylase large chain (Fragment)	0.451566952	0.545584045	1.208201892	0.000322803	PS
90	G8EEM9	Ribulose bisphosphate carboxylase large chain (Fragment)	0.439224459	0.530201342	1.20713073	0.000555879	PS
91	A0A1W6BQF9	Ribulose bisphosphate carboxylase large chain (Fragment)	0.400611621	0.482415902	1.204198472	0.001924496	PS
92	C6G1P2	Ribulose bisphosphate carboxylase large chain (Fragment)	0.556515479	0.669546436	1.203104786	0.002086768	PS
93	I2BGM7	Ribulose bisphosphate carboxylase large chain (Fragment)	0.500379651	0.600607441	1.20030349	0.000464902	PS
94	A0A142I7A5	Ribulose bisphosphate carboxylase large chain (Fragment)	0.339667458	0.406175772	1.195804197	0.021465427	PS
95	A0A0F6TFY2	Ribulose bisphosphate carboxylase large chain (Fragment)	0.511685117	0.611316113	1.194711538	0.013637455	PS

96	Q71N78	Ribulose bisphosphate carboxylase large chain (Fragment)	0.37914024	0.451726568	1.191449813	0.00183568	PS
97	I6N589	Ribulose bisphosphate carboxylase large chain (Fragment)	0.462271062	0.55018315	1.190174325	0.005510423	PS
98	Q95CF8	Ribulose bisphosphate carboxylase large chain (Fragment)	0.194563662	0.229613734	1.18014706	0.00219213	PS
99	I2E962	Ribulose bisphosphate carboxylase large chain (Fragment)	0.29856387	0.349962207	1.172151898	0.019082055	PS
100	E0D9L7	Ribulose bisphosphate carboxylase large chain (Fragment)	0.496212121	0.581439394	1.171755724	0.015642069	PS
101	K4EPE7	Ribulose bisphosphate carboxylase large chain (Fragment)	0.472927242	0.54822335	1.15921288	0.047687033	PS
102	Q37347	Ribulose bisphosphate carboxylase large chain (Fragment)	0.5	0.578680203	1.157360406	0.019157845	PS
103	A0A1C9HFU8	Ribulose bisphosphate carboxylase large chain (Fragment)	0.24691358	0.285493827	1.156249998	0.049712954	PS
104	P94046	Ribulose bisphosphate carboxylase large chain (Fragment)	0.392405063	0.453586498	1.155913977	0.04755552	PS
105	I2E906	Ribulose bisphosphate carboxylase large chain (Fragment)	0.450491308	0.520030234	1.154362416	0.021588609	PS
106	A0A251T1H4	Putative photosystem I reaction center subunit III protein	0.066666666	0.076811594	1.152173921	0.035168451	PS
107	W9RXI1	Glycerate dehydrogenase	0.101254481	0.116487455	1.150442476	0.028998039	PS
108	A0A0K9P513	Phosphoglycerate kinase	0.094941095	0.108108108	1.138686125	0.001991489	PS
109	A0A125R6I8	Ribulose bisphosphate carboxylase large chain (Fragment)	0.300595238	0.339285714	1.128712869	0.030375065	PS
110	Q7YJM6	Ribulose bisphosphate carboxylase large chain (Fragment)	0.456023651	0.513673319	1.126418153	0.006464558	PS
111	A0A078I0P1	Phosphoglycerate kinase	0.127777778	0.14375	1.125000001	0.019416076	PS
112	A0A120KLC1	Ribulose bisphosphate carboxylase large chain (Fragment)	0.596899225	0.670542636	1.123376623	0.007776836	PS
113	Q71N81	Ribulose bisphosphate carboxylase large chain (Fragment)	0.383882784	0.43003663	1.120229009	0.037409774	PS
114	X2D2J7	Ribulose bisphosphate carboxylase large chain (Fragment)	0.392055268	0.436096719	1.112334802	0.02427988	PS
115	A0A1D5AHD9	Ribulose bisphosphate carboxylase large chain (Fragment)	0.562028047	0.624595469	1.111324377	0.023950581	PS
116	A5BVF4	Uncharacterized protein	0.208333334	0.228632478	1.097435894	0.035403947	PS
117	A0A076GVP1	Ribulose-1,5-bisphosphate carboxylase/oxygenase large unit (Fragment)	0.398527865	0.431125132	1.081794196	0.025679186	PS

118	A0A200PMV3	Phosphoglycerate kinase	0.133744856	0.143347051	1.071794871	0.032532525	PS
119	A0A1S2XIN0	Phosphoglycerate kinase	0.136395268	0.14335421	1.051020407	0.024110109	PS
120	A0A200PYZ1	ATPase	0.098039215	0.083778966	0.85454546	0.000347891	PS
121	A0A1R3GDQ7	Photosystem I PsaN, reaction centre subunit N	0.159844054	0.136452242	0.853658538	0.032677925	PS
122	A0A0A7LIM5	Cytochrome f	0.089026915	0.064182194	0.720930227	0.005820686	PS
123	A0A151U9E4	Uncharacterized protein	0.016414141	0.031565656	1.923076919	0.013235603	redox
124	K8ECB3	Thioredoxin	0.044973545	0.082010582	1.823529391	0.00331976	redox
125	A0A2G5DW13	Uncharacterized protein	0.043478261	0.061594203	1.416666677	0.007490432	redox
126	W9QII5	Peroxiredoxin Q	0.026604069	0.037558685	1.411764713	0.024896162	redox
127	A0A2G5C8G0	Uncharacterized protein	0.175115207	0.210445469	1.201754389	0.033231258	redox
128	A0A2I0WJ74	Glutathione reductase, chloroplastic	0.010600707	0.008244994	0.777777746	0.016130067	redox
129	A0A1U8LIR6	2-methyl-6-phytyl-1,4-hydroquinone methyltransferase, chloroplastic-like isoform X2	0.016091954	0.022988506	1.428571455	0.0132356	secondary metabolism

[a] Protein ID, according to UniProtKB/Swiss-Prot database

[d] Function, protein function categorized using MapMan bin codes. ETC, mitochondrial electron transport chains; C1, one-carbon; TCA, tricarboxylic acid; CHO, carbohydrate; OPP, oxidative pentose phosphate.

Table S9. List of Proteins Identified in Leaf of *Clematis terniflora* DC. after HUV-B+D

No.	Protein ID[a]	Description	VV	VC	Fold changs	P-value	Annotation
1	I1MBR7	UTP--glucose-1-phosphate uridylyltransferase	0.009950249	0.019189765	1.92857137	0.031375739	glycolysis
2	A0A067L6G5	Uncharacterized protein	0.020043573	0.02788671	1.39130433	0.003125589	amino acid metabolism
3	C0PRV0	Lactoylglutathione lyase	0.02739726	0.03652968	1.33333333	0.019441768	Biodegradation of Xenobiotics
4	A0A2G5F096	Uncharacterized protein	0.01996008	0.027944112	1.4	0.047420656	development
5	G3EXN4	Enolase	0.026431718	0.04185022	1.58333333	0.003738445	glycolysis
6	A0A200RAD1	UTP--glucose-1-phosphate uridylyltransferase	0.019027484	0.028188866	1.48148151	0.031375734	glycolysis
7	Q42908	2,3-bisphosphoglycerate-independent phosphoglycerate mutase	0.011329756	0.016100179	1.42105262	0.03902052	glycolysis
8	A0A2I0XF37	Phosphoglycerate kinase, cytosolic	0.10556941	0.133000831	1.25984251	0.016105456	glycolysis
9	I1MQ89	Uncharacterized protein	0.070411985	0.088389513	1.25531915	0.005820687	glycolysis
10	A0A2G5D2H5	Phosphoglycerate kinase	0.138613861	0.170792079	1.23214286	0.009342662	glycolysis
11	A0A067F0Y6	Uncharacterized protein	0.055430711	0.068164794	1.22972973	0.028998039	glycolysis
12	A0A022R8A7	Uncharacterized protein	0.054054054	0.066066066	1.22222222	0.007182331	glycolysis
13	A0A1J3IGP7	Fructose-bisphosphate aldolase (Fragment)	0.032976827	0.039215686	1.18918919	0.035168443	glycolysis
14	A0A2G5DIJ2	Fructose-bisphosphate aldolase	0.077281192	0.091247672	1.18072289	0.042315657	glycolysis

15	A0A2I4G6H5	enolase 1	0.089339339	0.102102102	1.14285714	0.028998045	glycolysis
16	A0A068V643	Uncharacterized protein	0.058558559	0.066066066	1.12820512	0.007490439	glycolysis
17	K7KB09	Uncharacterized protein	0.033840948	0.043993232	1.29999999	0.0132356	hormone metabolism
18	K4BW79	2-methylene-furan-3-one reductase	0.057560137	0.074742268	1.29850747	0.003198203	misc
19	A0A0D6QRT0	ATP synthase subunit beta	0.045534151	0.053123176	1.16666666	0.022645999	mitochondrial electron transport / ATP synthesis
20	A0A2G5F6R8	ATP synthase subunit beta	0.054567022	0.061684461	1.13043479	0.043659462	mitochondrial electron transport / ATP synthesis
21	B9T1V8	ATP synthase subunit beta	0.055258467	0.062388592	1.12903226	0.047420636	mitochondrial electron transport / ATP synthesis
22	A0A0B0MQT9	Glutamine synthetase	0.052469136	0.037037037	0.70588235	0.00516687	N-metabolism.ammonia metabolism
23	C5YTC0	Uncharacterized protein	0.019965278	0.027777778	1.39130435	0.003125586	not assigned
24	W1P8B5	Uncharacterized protein	0.010007698	0.01308699	1.30769234	0.047420656	OPP
25	A0A0D3B1C7	Uncharacterized protein	0.018837019	0.024570025	1.30434782	0.02489617	OPP
26	A0A1J3H2G2	Ubiquitin-NEDD8-like protein RUB1 (Fragment)	0.267973856	0.375816993	1.40243902	0.006727071	protein
27	D7M7T7	Predicted protein	0.056661562	0.075038285	1.32432432	0.000124726	protein
28	C0PQG8	Elongation factor Tu	0.020179372	0.025411061	1.25925925	0.02489617	protein
29	A0A0D2SL22	Uncharacterized protein	0.029718876	0.036947791	1.24324323	0.00312559	protein
30	W9QWD3	RuBisCO large subunit-binding protein subunit beta	0.03857824	0.046380581	1.20224719	0.033471748	protein
31	A0A2H5P4R5	Uncharacterized protein	0.050531915	0.057624113	1.14035087	0.039020538	protein
32	W1PKM0	Uncharacterized protein	0.019680197	0.034440344	1.74999998	0.013235598	PS
33	A0A251VJH6	Putative chlorophyll A-B binding protein	0.026717557	0.043256998	1.61904764	0.014720594	PS
34	A0A2H5NKV2	Uncharacterized protein	0.135566188	0.218500798	1.61176471	0.008860642	PS
35	Q7M1Y7	Photosystem II oxygen-evolving complex protein 2 (Fragment)	0.207207207	0.324324324	1.56521739	0.007966202	PS
36	M5W912	Ferredoxin--NADP reductase	0.041208791	0.062271062	1.51111111	0.00096397	PS
37	J3MAL0	Ferredoxin--NADP reductase	0.039814815	0.058333333	1.46511627	0.001641289	PS
38	M4CGU3	Uncharacterized protein	0.015873016	0.023088023	1.45454549	0.0241101	PS
39	A0A0L9UT98	Uncharacterized protein	0.040639269	0.056621005	1.39325845	0.006185017	PS
40	W9RXI1	Glycerate dehydrogenase	0.079749104	0.110215054	1.38202248	0.003016383	PS
41	A0A2C9VVI6	Uncharacterized protein	0.041499331	0.057117359	1.37634409	0.001305458	PS
42	Q1EP00	Chlorophyll a-b binding protein, chloroplastic	0.047979798	0.065656566	1.36842105	0.027390718	PS
43	W9SCQ6	Ferredoxin--NADP reductase	0.051353875	0.070028011	1.36363636	0.003198202	PS
44	W8TP69	Glycerate dehydrogenase-like protein	0.043177893	0.058721934	1.36000001	0.019890752	PS

45	A0A0K9P513	Phosphoglycerate kinase	0.076230076	0.103257103	1.35454545	0.011294924	PS
46	G7IT85	Phosphoglycerate kinase	0.094641614	0.125956854	1.33088235	0.01643572	PS
47	Q19U04	NADH-dependent hydroxypyruvate reductase (Fragment)	0.111111111	0.147414741	1.32673267	0.001797912	PS
48	D2XUU3	Chloroplast managanese stabilizing protein (Fragment)	0.168446026	0.223013048	1.32394366	0.040165433	PS
49	A0A0A0KBL8	Uncharacterized protein	0.029082774	0.038478747	1.32307692	0.001756609	PS
50	A5BVF4	Uncharacterized protein	0.16025641	0.211538461	1.32	0.016691992	PS
51	A0A151UC86	Uncharacterized protein	0.049421661	0.065194532	1.31914894	0.011912536	PS
52	A0A200QG47	Aminotransferase	0.047381546	0.062344139	1.31578946	0.021311643	PS
53	M4QSX4	Transketolase	0.03480589	0.045515395	1.30769232	0.004812677	PS
54	A0A2H5NQP8	Uncharacterized protein	0.080614923	0.104611924	1.29767442	0.009711821	PS
55	A0A200PYZ1	ATPase	0.084670232	0.109625668	1.29473684	0.008635793	PS
56	A1BQW9	Transketolase (Fragment)	0.05	0.064444444	1.28888889	0.002890007	PS
57	A0A251VGE5	Putative photosystem I PsaA/PsaB	0.0486618	0.062530414	1.28500001	0.03003475	PS
58	A0A0E0A154	Phosphoglycerate kinase	0.156378601	0.200823045	1.28421053	0.020204207	PS
59	A0A218X6H3	Glyceraldehyde-3-phosphate dehydrogenase	0.091584159	0.116336634	1.27027027	0.037590112	PS
60	A0A2G5ESQ6	Phosphoribulokinase	0.129084967	0.161764706	1.25316456	0.002364389	PS
61	A0A118K159	Phosphoglycerate kinase	0.057122198	0.071583514	1.25316456	0.038029298	PS
62	A0A200R9X0	Transketolase	0.043010753	0.053315412	1.23958333	0.016896273	PS
63	A0A1R3K0M1	Uncharacterized protein	0.028635347	0.035346756	1.23437501	0.010636625	PS
64	A0A2I4DTC0	peroxisomal (S)-2-hydroxy-acid oxidase GLO1	0.047229791	0.058128974	1.23076923	0.022394204	PS
65	A0A0F7G6G0	Chlorophyll a-b binding protein, chloroplastic (Fragment)	0.125786164	0.154716981	1.23	0.036174812	PS
66	A0A059P277	Photosystem II D2 protein	0.099150142	0.121813031	1.22857143	0.006352257	PS
67	K4BP59	Glyceraldehyde-3-phosphate dehydrogenase	0.106934002	0.131161237	1.2265625	0.046693164	PS
68	A0A1J7HK61	Uncharacterized protein	0.045413261	0.055404178	1.22	0.023399371	PS
69	A0A2H3Z4Q2	Glycine cleavage system P protein	0.009294872	0.011217949	1.20689656	0.013235623	PS
70	M0TFM8	Uncharacterized protein	0.055404178	0.066303361	1.19672131	0.022394204	PS
71	A0A1B2LUN5	Glyceraldehyde-3-phosphate dehydrogenase	0.130833333	0.155833333	1.1910828	0.033535409	PS
72	A0A2G5EUG6	Chlorophyll a-b binding protein, chloroplastic	0.09122807	0.107602339	1.17948718	0.048811551	PS
73	H2KWC8	Chlorophyll a-b binding protein, chloroplastic	0.069892473	0.082437276	1.17948718	0.024896163	PS

74	A0A0A7LEE5	Photosystem II CP47 reaction center protein	0.101049869	0.118766404	1.17532467	0.016983257	PS
75	A0A0A0L177	Uncharacterized protein	0.050970874	0.05987055	1.17460318	0.005328129	PS
76	A0A1J7IIL7	Glyceraldehyde-3-phosphate dehydrogenase	0.064301552	0.075388027	1.1724138	0.01794791	PS
77	K4KJF0	ATP synthase subunit alpha, chloroplastic (Fragment)	0.093439364	0.109343936	1.17021277	0.013942331	PS
78	M1AH35	Chlorophyll a-b binding protein, chloroplastic	0.077595628	0.090710383	1.16901409	0.022394202	PS
79	C0J9P4	ATP synthase subunit beta (Fragment)	0.308614232	0.359550562	1.16504854	0.008074424	PS
80	A0A1I7PIS3	ATP synthase subunit beta (Fragment)	0.30713246	0.355895196	1.15876777	0.01068163	PS
81	A0A059TLB1	Photosystem II 32 kDa protein (Fragment)	0.307843137	0.35490196	1.15286624	0.022394207	PS
82	A0A0M3LBA7	ATP synthase subunit beta, chloroplastic	0.327333333	0.377333333	1.15274949	0.010837472	PS
83	B7S4V7	ATP synthase subunit beta (Fragment)	0.249457701	0.287057122	1.15072464	0.004557179	PS
84	A0A0A7DB11	ATP synthase subunit beta (Fragment)	0.194829178	0.219759926	1.12796209	0.026559888	PS
85	Q9MRM1	ATP synthase subunit beta (Fragment)	0.204868154	0.23056119	1.12541254	0.04311073	PS
86	A0A1R3GDQ7	Photosystem I PsaN, reaction centre subunit N	0.157894737	0.167641325	1.06172839	0.007490434	PS
87	A0A291L206	Ribulose bisphosphate carboxylase large chain (Fragment)	0.548562548	0.529137529	0.96458924	0.012771796	PS
88	G8EEM9	Ribulose bisphosphate carboxylase large chain (Fragment)	0.51901566	0.499627144	0.96264368	0.014720594	PS
89	B5L2V5	Ribulose bisphosphate carboxylase large chain (Fragment)	0.547901821	0.525732383	0.95953757	0.025514501	PS
90	Q8WJX3	Ribulose bisphosphate carboxylase large chain (Fragment)	0.486142322	0.459925093	0.94607088	0.041635386	PS
91	E5G0I9	Ribulose bisphosphate carboxylase large chain (Fragment)	0.54082397	0.507865169	0.93905817	0.047983364	PS
92	Q9GD27	Ribulose bisphosphate carboxylase large chain (Fragment)	0.373304782	0.346181299	0.92734226	0.007090812	PS
93	B0L5K7	Ribulose bisphosphate carboxylase large chain (Fragment)	0.284552845	0.262379897	0.92207792	0.016010967	PS
94	K8ECB3	Thioredoxin	0.05026455	0.082010582	1.63157894	0.032677923	redox
95	I6P9I5	Cytosolic ascorbate peroxidase (Fragment)	0.029513889	0.043402778	1.47058823	0.02321516	redox
96	A0A1J6I7J0	2-cys peroxiredoxin bas1, chloroplastic	0.053682897	0.072409488	1.34883721	0.006073612	redox
97	A0A1D8H339	2-Cys peroxiredoxin	0.072727272	0.093333333	1.28333334	0.009205887	redox
98	A0A2G5C8G0	Uncharacterized protein	0.153609831	0.19047619	1.24	0.032677923	redox
99	A0A2G5CER3	Uncharacterized protein (Fragment)	0.009569378	0.014354067	1.50000005	0.025721415	signalling
100	F6HCT7	Uncharacterized protein	0.009775171	0.015151515	1.54999994	0.007933093	stress
101	A9RCV9	Predicted protein	0.046296296	0.064814815	1.4	0.001418611	stress

102	A9S0A3	Predicted protein	0.048868313	0.067386831	1.37894737	0.00168533	stress
103	A0A251UZB2	Putative heat shock protein 70 family	0.051440329	0.069958848	1.36	0.00168533	stress
104	A0A0K9Q3W1	70 kDa heat shock protein	0.055214724	0.074642127	1.35185185	0.009716249	stress
105	A0A0K9P9U3	Heat shock cognate 70 kDa protein	0.051162791	0.06873385	1.34343434	0.003016383	stress
106	A0A1D1XZB0	Heat shock cognate protein (Fragment)	0.061660561	0.082417582	1.33663367	0.001050578	stress
107	A0A1D1Y9S2	Heat shock protein 4 (Fragment)	0.057563588	0.076305221	1.32558139	0.002192129	stress
108	C9WCK6	Heat shock protein 70	0.044034818	0.057859703	1.3139535	0.006107226	stress
109	D8RBE2	Uncharacterized protein	0.04718417	0.061897514	1.31182797	0.00539689	stress
110	A0A165A740	Uncharacterized protein	0.065769806	0.085949178	1.30681818	0.003795103	stress
111	A0A1S3YP64	luminal-binding protein 4-like	0.037481259	0.048975512	1.30666666	0.030337359	stress
112	A0A0D2Q3K9	Uncharacterized protein	0.03862661	0.050071531	1.29629629	0.004812681	stress
113	A0A1S3E0E2	heat shock cognate 70 kDa protein 2-like isoform X3	0.054095827	0.070066976	1.29523809	0.000606962	stress
114	A0A022PYG3	Uncharacterized protein	0.050102249	0.064417178	1.28571429	0.000898386	stress
115	A0A2H3ZD96	heat shock cognate 70 kDa protein 2-like	0.050411523	0.064814815	1.28571429	0.011446916	stress
116	A0A251SC78	Putative heat shock cognate 70 kDa protein 2	0.057524397	0.073959938	1.28571428	0.005370443	stress
117	S8EAM3	Heat shock protein hsp70 (Fragment)	0.025228126	0.032206119	1.27659576	0.002890007	stress
118	A0A1J3FXE7	Putative mediator of RNA polymerase II transcription subunit 37e	0.057859703	0.073732719	1.27433629	0.005097541	stress
119	M5W3N4	Uncharacterized protein	0.05984252	0.076115485	1.27192981	0.00149353	stress
120	I1IHD0	Uncharacterized protein	0.063271605	0.080246914	1.26829268	0.000175635	stress
121	A0A2G5EBY1	Uncharacterized protein	0.063687725	0.080636878	1.26612904	0.000122704	stress
122	B7ZZ42	Heat shock 70 kDa protein 3	0.055983565	0.070878274	1.26605504	0.000131532	stress
123	A0A0D9VSI0	Uncharacterized protein	0.05025641	0.063589744	1.26530613	0.001430013	stress
124	A0A1D6KE29	Heat shock protein 70	0.054442733	0.068310221	1.25471697	0.000672594	stress
125	M0TS86	Uncharacterized protein	0.052983539	0.066358025	1.25242719	0.001192882	stress
126	G7KWU8	Heat shock cognate 70 kDa protein	0.061213992	0.076646091	1.25210085	0.001312729	stress
127	B9NBF4	Heat shock protein 70 cognate	0.056584362	0.070473251	1.24545455	0.008820839	stress
128	A0A251UNC3	Putative heat shock protein	0.058127572	0.072016461	1.23893805	0.002811398	stress
129	J7EM74	HSP70	0.062564103	0.077435897	1.23770491	0.00149311	stress
130	A0A1S4B2J7	heat shock cognate 70 kDa protein 2	0.047839506	0.058641975	1.22580645	0.001016663	stress
131	A0A200Q738	Heat shock protein 70 family	0.042012162	0.051409619	1.22368421	0.001050578	stress
132	A0A2G5EQF7	Uncharacterized protein	0.051428571	0.062857143	1.22222222	0.003448384	stress
133	A0A1D5XSS6	Uncharacterized protein	0.060699589	0.074074074	1.22033898	0.018562566	stress
134	A0A1S2YJT8	heat shock cognate 70 kDa protein 2	0.051874679	0.063174114	1.21782177	0.002243306	stress

135	A0A251VHZ2	Putative heat shock protein 70 (Hsp 70) family protein	0.054192229	0.06595092	1.21698113	0.008023124	stress
136	A0A140CZC8	Heat shock cognate 70 kDa protein 2-like protein	0.061308604	0.073673364	1.20168068	0.003035809	stress
137	W1NRJ1	Uncharacterized protein	0.059360731	0.071029934	1.19658119	0.010030893	stress
138	A0A0E0JVK4	Uncharacterized protein	0.057098766	0.067901235	1.18918919	0.010163647	stress
139	H1ZXA8	Heat shock protein 70 isoform 2	0.066252588	0.078674949	1.18750001	0.000427093	stress
140	A0A2H3YRJ8	heat shock cognate 70 kDa protein 2-like	0.044238683	0.052469136	1.18604652	0.001323896	stress
141	A0A2C9V2W2	Uncharacterized protein	0.062242798	0.073559671	1.18181818	0.002680097	stress
142	A0A0G3B490	Heat shock protein 70	0.057259714	0.067484663	1.17857143	0.010119689	stress
143	A0A0B2R5Y0	Heat shock cognate 70 kDa protein 2	0.061119671	0.071905495	1.17647058	0.007015543	stress
144	A0A0F7CS07	Heat shock cognate 70 kDa-like protein (Fragment)	0.042281219	0.049655851	1.17441861	0.028460205	stress
145	C5XPN2	Uncharacterized protein	0.058127572	0.067901235	1.16814159	0.019103658	stress
146	M5X5S2	Uncharacterized protein	0.062146893	0.072419106	1.16528925	0.01011969	stress
147	B2D2G5	70 kDa heat shock protein	0.055469954	0.064201335	1.1574074	0.022269885	stress
148	A0A2C9VM37	Uncharacterized protein	0.062757202	0.072530864	1.1557377	0.012072549	stress
149	C4J410	Heat shock 70 kDa protein	0.058127572	0.066872428	1.15044248	0.019082048	stress
150	A0A067L994	Uncharacterized protein	0.059670782	0.068415638	1.14655173	0.006858968	stress
151	A0A067LJW8	Uncharacterized protein	0.072393301	0.082117774	1.13432835	0.003125589	stress
152	A0A2G5DAJ3	Carbonic anhydrase	0.032835821	0.044776119	1.36363636	0.036277821	TCA / org

[a] Protein ID, according to UniProtKB/Swiss-Prot database

[d] Function, protein function categorized using MapMan bin codes. ETC, mitochondrial electron transport chains; C1, one-carbon; TCA, tricarboxylic acid; CHO, carbohydrate; OPP, oxidative pentose phosphate.
